# Nitrate Reduction Functional Genes and Nitrate Reduction Potentials Persist in Deeper Estuarine Sediments. Why?

**DOI:** 10.1371/journal.pone.0094111

**Published:** 2014-04-11

**Authors:** Sokratis Papaspyrou, Cindy J. Smith, Liang F. Dong, Corinne Whitby, Alex J. Dumbrell, David B. Nedwell

**Affiliations:** School of Biological Sciences, University of Essex, Wivenhoe Park, Colchester, United Kingdom; German Cancer Research Center, Germany

## Abstract

Denitrification and dissimilatory nitrate reduction to ammonium (DNRA) are processes occurring simultaneously under oxygen-limited or anaerobic conditions, where both compete for nitrate and organic carbon. Despite their ecological importance, there has been little investigation of how denitrification and DNRA potentials and related functional genes vary vertically with sediment depth. Nitrate reduction potentials measured in sediment depth profiles along the Colne estuary were in the upper range of nitrate reduction rates reported from other sediments and showed the existence of strong decreasing trends both with increasing depth and along the estuary. Denitrification potential decreased along the estuary, decreasing more rapidly with depth towards the estuary mouth. In contrast, DNRA potential increased along the estuary. Significant decreases in copy numbers of 16S rRNA and nitrate reducing genes were observed along the estuary and from surface to deeper sediments. Both metabolic potentials and functional genes persisted at sediment depths where porewater nitrate was absent. Transport of nitrate by bioturbation, based on macrofauna distributions, could only account for the upper 10 cm depth of sediment. A several fold higher combined freeze-lysable KCl-extractable nitrate pool compared to porewater nitrate was detected. We hypothesised that his could be attributed to intracellular nitrate pools from nitrate accumulating microorganisms like *Thioploca* or *Beggiatoa*. However, pyrosequencing analysis did not detect any such organisms, leaving other bacteria, microbenthic algae, or foraminiferans which have also been shown to accumulate nitrate, as possible candidates. The importance and bioavailability of a KCl-extractable nitrate sediment pool remains to be tested. The significant variation in the vertical pattern and abundance of the various nitrate reducing genes phylotypes reasonably suggests differences in their activity throughout the sediment column. This raises interesting questions as to what the alternative metabolic roles for the various nitrate reductases could be, analogous to the alternative metabolic roles found for nitrite reductases.

## Introduction

Increased anthropogenic inputs of nitrogen (N) from fertiliser run-off, sewage discharges and aquaculture into coastal systems, like estuaries, stimulate primary production (eutrophication), occasionally leading to anoxia in the water column and mass mortality of fish stocks and other macrofauna [1]. Benthic microbial processes such as denitrification can alleviate the effect of increased N loads, removing up to 50% of the N load in many estuaries as N_2_ or N_2_O [2], [3]. Anaerobic ammonium oxidation (Anammox) may also remove significant amounts of nitrite and ammonium as N_2_ at some marine and estuarine sites [4], [5]. However, another process, dissimilatory nitrate reduction to ammonium (DNRA) converts nitrate to biologically available ammonium, which can be retained within the system.

Denitrification and DNRA occur simultaneously under oxygen-limited or anaerobic conditions and compete for nitrate and organic carbon. The first step in both denitrification and DNRA is nitrate reduction to nitrite, catalysed by one of two nitrate reductase enzymes; membrane bound NAR or NAP that is located in the periplasm. In nitrate denitrifiers, NAR is expressed predominately under anaerobic denitrifying conditions, and NAP under aerobic conditions [6]. NAR has been shown to be most effective in nitrate ammonifiers under high nitrate conditions, and NAP under low nitrate conditions [7]. Expression of NAP is also higher when a more reduced carbon source is available for bacterial growth [8]. The next step in the two processes is distinct and for denitrification involves the enzyme nitrite reductase (NIR) converting nitrite to nitric oxide, and for DNRA the nitrite reductase (NRF) enzyme which converts nitrite to ammonium. Thus, the environmental abundance and balance of activity of these two functional groups of nitrate respiring populations (i.e. denitrification and DNRA bacteria) in estuarine sediments depends on factors such as labile organic carbon and nitrate availability, the ratio of electron donor/acceptor (carbon∶nitrate), sulfide concentration, and temperature [1], [9], [10]. Therefore, understanding the mechanisms that control competition between the two nitrate reducing groups is important in controlling their ecological activity and the fate of N load in natural ecosystems.

The Colne estuary (UK) is a macrotidal, hyper-nutrified, muddy estuary with strong gradients of nitrate and ammonium from inputs from the river and a sewage treatment plant at the estuary head. In the Colne, 20–25% of the total N load entering the estuary is removed by denitrification, with highest rates at the estuary head decreasing towards the mouth [11]–[13]. Gene sequences related to the enzymes involved in denitrification and DNRA (*napA*, *narG*, *nirK*, *nirS*, *nosZ*, *nrfA*) have been isolated from these systems and have been shown to differ significantly from previously recorded sequences [14], [15]. In addition, gene copy number in surface sediments significantly decline from the estuary head towards the estuary mouth. Despite their ecological importance, there has been little investigation of how denitrification and DNRA related genes vary vertically with sediment depth.

We hypothesise that a decrease in the concentrations of electron acceptors (nitrate and nitrite) and organic carbon along an estuarine gradient (and with sediment depth) would result in differences in the distribution of key functional genes and that these differences would be related to the relative magnitudes of the capacities of the corresponding N processes. To test these hypotheses we: (1) measured nitrate reduction potential (NRP) rates both laterally along the estuary and vertically with sediment depth, (2) estimated the contribution of potential denitrification compared to DNRA, (3) estimated the contribution of NAR and NAP to the potential of nitrate reduction processes, and (4) related these potentials to the abundance of genes related to nitrate (*narG*, *napA*) and nitrite (*nirS* and *nrfA*) reduction.

## Materials and Methods

### Site description

Sediment cores were collected in May–June 2007 using plexiglass tubes (8 cm internal diameter×40 cm length) from the head of the Colne estuary at the Hythe (51°52′41.6″N, 0°55 59.4E), midway down the estuary at Alresford (51°50′32.4″N, 0°58′53.6″E), and from the estuary mouth at Brightlingsea (51°48′22.4″N, 1°0′36.6″E). No specific permissions were required for sampling at these locations according to current UK law and no harm was caused to any endangered or protected species. Sediment cores were immediately put on ice, returned to the laboratory within 1 h of sampling, and kept at 4°C until further processing. Depending on tidal state, salinity ranged between 2–17, (Hythe), 20–32, (Alresford) and 28–32 (Brightlingsea) [13].

### Nitrate reduction potentials

#### Slurry preparation

All slurry experiments were performed within a maximum of two days from sediment core collection. Between 8–10 cores were sliced at 0–1, 3–4, 6–8 and 18–20 cm depths and slices from the same depth were pooled. Sediment slurries (50% v/v) from each depth were prepared by homogenizing the sediment with anaerobic artificial seawater [16] at the corresponding salinity of each site. Equal volumes (30 mL) of slurry were dispensed within an anaerobic glove bag into 60 mL bottles fitted with butyl rubber caps. The bottles were sealed and flushed with N_2_ for 15 min.

#### Nitrate reduction kinetics

A sodium nitrate solution (100 mM) was added to a series of slurries from each sediment depth to obtain initial nominal concentrations of 0, 0.5, 1, 2, or 5 mM nitrate. After measuring initial concentrations in six bottles, triplicate bottles from each depth and each nitrate concentration were incubated (3 h, 20°C) on a rocking platform at 70 rev min^−1^ (STR6, Stuart Bibby, UK). The effect of organic donor availability was studied by adding sodium acetate (final concentration 10 mM) to another set of bottles at the highest nitrate concentration used.

From each bottle, 10 mL of sediment slurries were centrifuged (Harrier 15/80, MSE UK Ltd, 6 min, 5000× g), and the supernatant filtered through a 0.22 µm pore size filter and frozen (−20°C) for later determination of NO_3_^−^. Nitrate reduction potential (NRP) rates were calculated by the change in nitrate concentration with time between start and end. Preliminary experiments showed a linear decrease in concentration for up to 6 h (data not shown). Nitrate reduction kinetics were derived by least squares fitting a Michaelis-Menten rate expression to the NRP rates: V = V_max_ * [NO_3_^−^]/(K_m_+[ NO_3_^−^]), where V is nitrate reduction rate, K_m_ is the half saturation constant for NO_3_^−^ and V_max_ is the maximum rate.

#### Nitrate reduction pathways and NAR or NAP enzyme contribution

To a series of slurries from each sediment depth, acetylene was added to the headspace (10% v/v) to inhibit the reduction of N_2_O to N_2_ and thus provide a measurement of denitrification by comparing N_2_O accumulation levels in the presence and absence of acetylene [17], [18]. The addition of acetylene has been criticised due to among other problems the underestimation of denitrification; other methods such as the ^15^N addition method are increasingly used. However, for the measurement of potential rates and especially in areas with moderate or high NO_3_^−^ concentrations, the acetylene inhibition technique can validly be applied to compare between sites [19]. Chlorate was added (final concentration 20 mM) as a specific inhibitor of NAR applicable to sediment slurries [19]; in some bacterial cultures chlorate may only incompletely inhibit NAR [20], in which case our technique may give a conservative estimate of the contribution of NAR to nitrate reduction potential.

Slurries were pre-incubated (30 min, 20°C) on a rocking platform as described above. Then, nitrate was added to each bottle at a high initial concentration (Hythe: 5 mM, Alresford and Brightlingsea: 2 mM), as determined from the initial nitrate kinetic experiment, to maintain nitrate saturation during incubation. After determining initial nitrate concentrations, slurries were incubated (3 h, 20°C) on a rocking platform. To determine N_2_O concentration following incubation, 12 mL were taken from the headspace of each bottle with a hypodermic syringe and transferred to a 12 mL exetainer (Labco, UK). Slurries (20 mL) were processed as described above to later measure the concentrations of NO_3_^−^, NO_2_^−^, and NH_4_^+^ in the filtrates. The sediment pellet was frozen (−20°C), and then four sequential extractions were performed by adding 10 mL of 2 M KCl solution, the sediment incubated for 30 min at 4°C, vortexed every 10 min, centrifuged (6 min, 4000× g) and the supernatant collected (i.e. a total of 40 mL) to determine KCl-extractable plus freeze-lysable (KCl_ex_) NH_4_^+^. Initial trials showed that four sequential extractions were sufficient to recover >95% of the KCl extractable NH_4_^+^. Potential DNRA was calculated as the increase in total NH_4_^+^, assuming that nitrogen mineralization is uncoupled from the terminal carbon oxidation process [21].

### In situ sampling of functional genes and environmental variables

Triplicate sediment cores collected during emersion from each site were sliced at 0–1, 1–2, 2–3, 3–4, 4–5, 5–6, 6–8, 10–12, 14–16 and 18–20 cm intervals. To avoid any cross-contamination, only the centre of each slice was homogenized and samples for DNA extraction dispensed into sterile 1.5 mL tubes and stored at −80°C.

Another three cores from each site were sliced as above and used to determine density, water content, chlorophyll *a*, organic carbon and nitrogen and grain size distribution at each sediment depth. A sediment sample (∼2–3 g) was stored at −20°C to later determine KCl_ex_ nutrient pools using a 5 mL 2 M KCl solution. Porewater for the determination of nutrients (NO_3_^−^, NO_2_^−^, and NH_4_^+^) was collected by centrifuging (6 min, 4000× g) the remaining sediment.

Five cores were used for determination of macrofaunal abundance. The sediment was sieved over a 0.5 mm mesh, animals collected and preserved in 70% (v/v) ethanol with Rose Bengal until further identification into major taxonomic groups.

### Chemical analyses

NO_3_^−^ and NO_2_^−^ concentrations were measured spectrophotometrically on a segmented flow autoanalyser (Scan^plus^, Skalar Analytical B.V., The Netherlands). Ammonium was determined manually using the salicylate method [22]. N_2_O was measured with a gas chromatograph fitted with a ^63^Ni electron capture detector [11] and dissolved concentrations calculated according to Weiss and Price [23]. Density, porosity, and water content of the sediment and slurries were determined by weighing a known volume of wet sediment and then drying it at 60°C to constant weight. Chlorophyll *a* was determined spectrophotometrically after extraction with 100% methanol buffered with MgCO_3_ before and after acidification [24]. Organic carbon (C_org_) and total N was measured on a CHN analyzer [25]. Grain size distribution was determined according to Buchanan [26]. Biogeochemical data from the current work have been deposited at the Pangaea database (http://doi.pangaea.de/10.1594/PANGAEA.830237)

### Total DNA extraction

Nucleic acids were extracted by a combined mechanical-chemical extraction protocol as described in Smith et al [14]. Total extracted genomic DNA was then purified using a Sepharose 4B column to remove humic acids [27]. Sepharose 4B was packed by gravity in a 2.5 mL syringe to a final volume of 2.5 mL. The column was equilibrated with 4 vol high salt TE buffer (100 mM NaCl, 10 mM Tris, 1 mM EDTA; pH 8.0 with HCl). Crude DNA extract was added to the column followed by several additions of 250 µl high salt TE buffer. The eluate was collected in 250 µL fractions and each fraction was tested using bacterial 16S rRNA gene primers 1369F and Prok 1492R [28] (Table S1). One microlitre of RNA was added to a 50-µL PCR mixture containing 1× Qiagen PCR buffer (Qiagen), 1.5 mM MgCl2, 0.2 mM of each deoxynucleotide triphosphate (dNTP), 0.25 µM of each primer, and 2.5 units of Taq polymerase (Qiagen). The reaction mixture was initially denatured at 95°C for 5 min, followed by 30 cycles of 95°C for 30 s, annealing at 55°C for 30 s, and elongation at 72°C for 30 s, followed by a final extension step at 72°C for 5 min. Following PCR testing, the fractions of each eluate that gave a positive PCR result were pooled, concentrated following another cycle of precipitation with ethanol as described above, resuspended in 100 µL sterile MilliQ water, and frozen at −80°C.

### qPCR standards and analysis

We used a suite of qPCR primers and Taqman probes (Applied BioSystems, USA) designed to target the 16S rRNA gene [28], *napA*, *narG*, *nirS* and *nrfA* genes [14], i.e. three sets of primers for *napA* (*napA*-1, *napA*-2, *napA*-3), two for *narG* (*narG*-1, *narG*-2), three for *nirS* (*nirS*-e, *nirS*-m, *nirS*-n) and one for *nrfA* (*nrfA*-2) (Table S1). For each primer combination, qPCR assays for each gene were performed within a single assay plate using DNA standard curves constructed as described previously [14], [29], thus permitting direct comparison of absolute numbers between DNA samples. Each assay contained a standard curve containing 10^3^ to 10^8^ DNA amplicons µL^−1^ for amplification by qPCR, independent triplicate sediment DNA samples from each of the three sites along the Colne estuary, and triplicate no-template controls (NTC). qPCR amplification mixtures, protocols and final gene number calculations were performed as described previously with no modifications [14] using an ABI 7000 Sequence Detection System (Applied BioSystems).

### Pyrosequencing

Following the premise (see discussion) that the presence of nitrate reduction genes in deeper sediments where porewater nitrate was absent was due to nitrate-accumulating bacteria in the sediment, pyrosequencing analysis was conducted to examine if these organisms were present. Pyrosequencing was performed on triplicate DNA samples using a Roche 454 FLX instrument with Titanium reagents for tag-encoded FLX amplicon pyrosequencing (TEFAP) (Research and Testing Laboratory, Lubbock, Texas, USA, http://www.researchandtesting.com) based upon standard methods [30]. The 16S rRNA gene was PCR amplified using the primers Gray28F and Gray519R [31] (Table S1) and amplicon libraries analysed following a modification of the PANGEA pipeline [32]. All sequences (total raw sequences = 157,000) were checked for the presence of correct pyrosequencing adaptors, 10-bp barcodes and taxon-specific primers and any sequences containing errors in these primer regions were removed. In addition, sequences >200 bp in read length, sequences with low quality scores (<20), and sequences containing homopolymer inserts (maximum homopolymer length = 6 bp) were also removed from further analysis. All sequences were aligned using the (mega)Blast algorithm [33] against a non-redundant database of 16S rRNA sequences from cultured isolates in the RDP and Greengenes databases. Once reads matching known cultured isolates (95% sequence similarity) had been identified the remaining unidentified reads were clustered into operational taxonomic units (OTUs – 95% sequence similarity) using the UClust algorithm [34] and representative sequences from each OTU were assigned taxonomy using RDP classifier, a naïve Bayesian classifier [35]. Finally, all singletons were removed before further analysis [36]. The presence of *Thioploca spp.* (a known nitrate-accumulating bacteria) was further tested by aligning *Thioploca spp* 16S rRNA sequences (from GenBank) against all pyrosequencing reads using pairwise Needleman-Wunsch alignments. All raw sequence reads from each of the 24 amplicon libraries have been submitted to MG-RAST (http:/metagenomics.anl.gov) and are stored under the project name ‘nitrate reduction in estuarine sediments’ (http://metagenomics.anl.gov/linkin.cgi?project=7242), with accession numbers: 4547523.3–4547546.3.

### Statistical analysis

Best-fit Michaelis Menten curves of the rate data were obtained using the Sigmaplot 11.0 software. A two-way permutational analysis of variance (PERMANOVA) using Euclidean distances [37] was applied with each of measured rates, functional gene abundance and % contribution of rates as the response variable and site and depth as fixed factors. Percentages were arcsin(x) transformed. Functional gene abundances were ln(x+1) transformed to retain information regarding relative abundances but to reduce differences in scale among them [38]. With regard to the gene profiles in the sediment, because depth intervals within cores are not independent, core identity was introduced as a new random factor nested within site.

We investigated the relationship between potential rates from the slurry experiments with *in situ* functional gene abundance, C_org_ availability and C∶N ratio by performing distance based multiple regression [39], after removing environmental variables with correlation >0.9, using the best selection procedure and the AIC criterion. Finally, the relation of environmental variables with nitrate reduction functional gene assemblage was investigated using multivariate multiple regression as mentioned above on a Bray-Curtis dissimilarity matrix of ln(x+1) transformed functional gene variables. All analyses were obtained using PRIMER 6.0 for Windows [40] and the PERMANOVA+ add-on for PRIMER [37].

## Results and Discussion

### Kinetics of nitrate reduction

**Figure 1:**
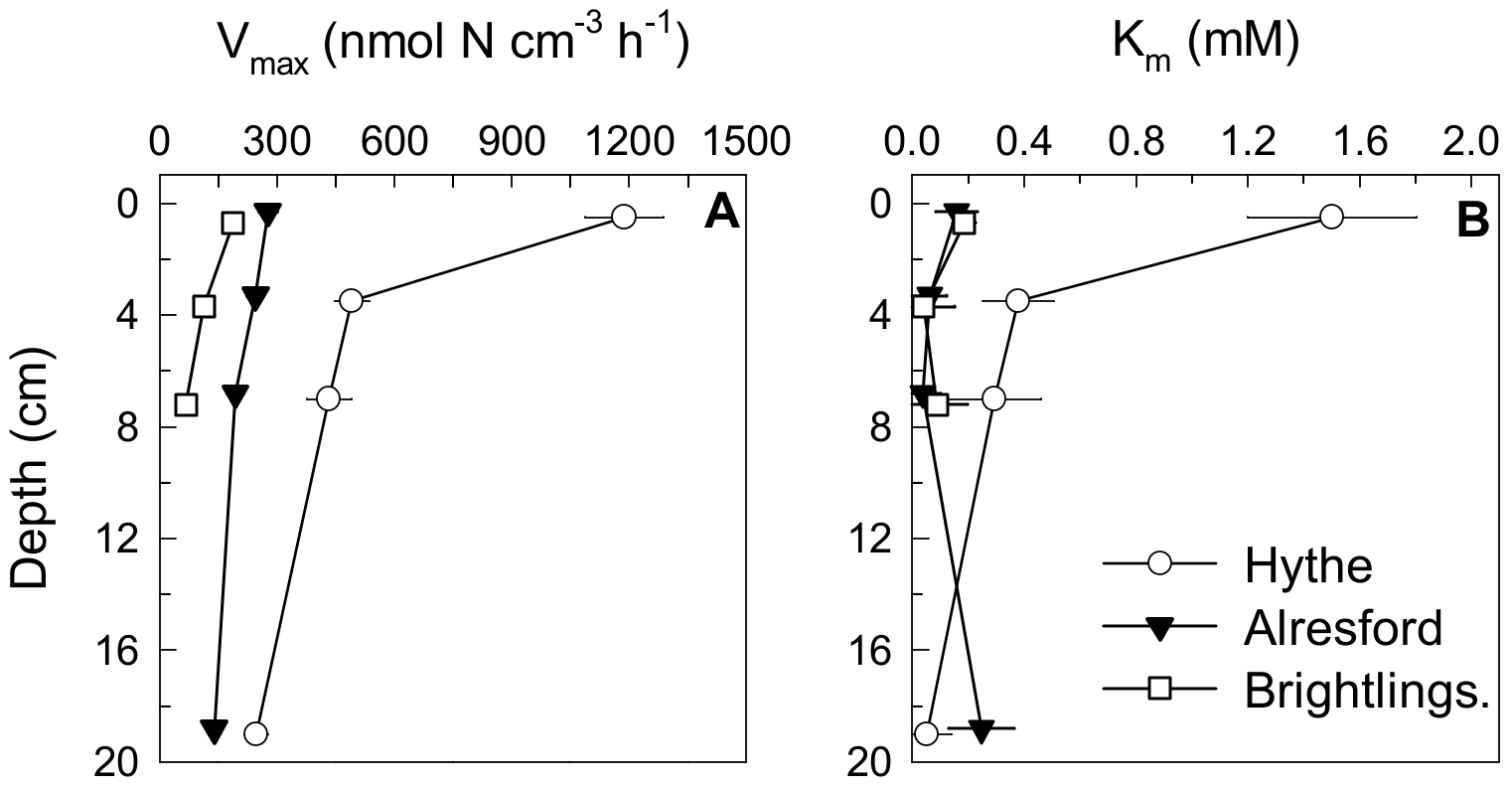
Vertical profiles of sediment nitrate reduction pathways potentials. (A) Nitrate reduction (NRP), (B) denitrification (DN), and (C) dissimilatory nitrate reduction to ammonium (DNRA) potentials, (D) contribution (%) to NRP by DN and (E) by DNRA and (F) contribution (%) of NAR based NRP from slurry experiments conducted with sediment from the Hythe, Alresford and Brightlingsea collected in June 2007. Data points have been offset by 0.2 cm to facilitate observation of error bars. Data are mean ±SE (n = 3).

The maximum estimated nitrate reduction rate values, V_max_, obtained in the slurries corresponded to the maximum nitrate-reducing activities the resident microbial populations could sustain with excess nitrate and the *in situ* availability of electron donors and other possible limiting factors such as nutrients. Application of the best fit of the Michaelis–Menten kinetics (Table S2) to the rate data revealed a decrease in the capacity (V_max_) for benthic nitrate reduction down the estuary, with highest values in surface sediment at Hythe (Fig. 1). The values of the half-saturation constants, K_m_, which give some measure of the affinity of the sediment microbial community for nitrate, showed highest values (i.e. lowest affinity) at the sediment surface at Hythe (Fig. 1). This means that at the Hythe, the sediment surface nitrate-reducing microbial community operated well below its maximum potential rates of nitrate reduction, as the nitrate concentrations usually found in the overlying water [12] are greatly below K_m_ values. In contrast, at Alresford and Brightlingsea, the K_m_ values were much lower (i.e. higher affinity for nitrate) than at the Hythe, with no noticeable differences of K_m_ with depth at each site, nor between the two sites, equating to the much lower nitrate concentrations available down the estuary towards the mouth. These low K_m_ values clearly indicate adaptation of the nitrate-utilising community to better scavenge nitrate at low nitrate concentrations.

### Nitrate reduction pathways

**Figure 2:**
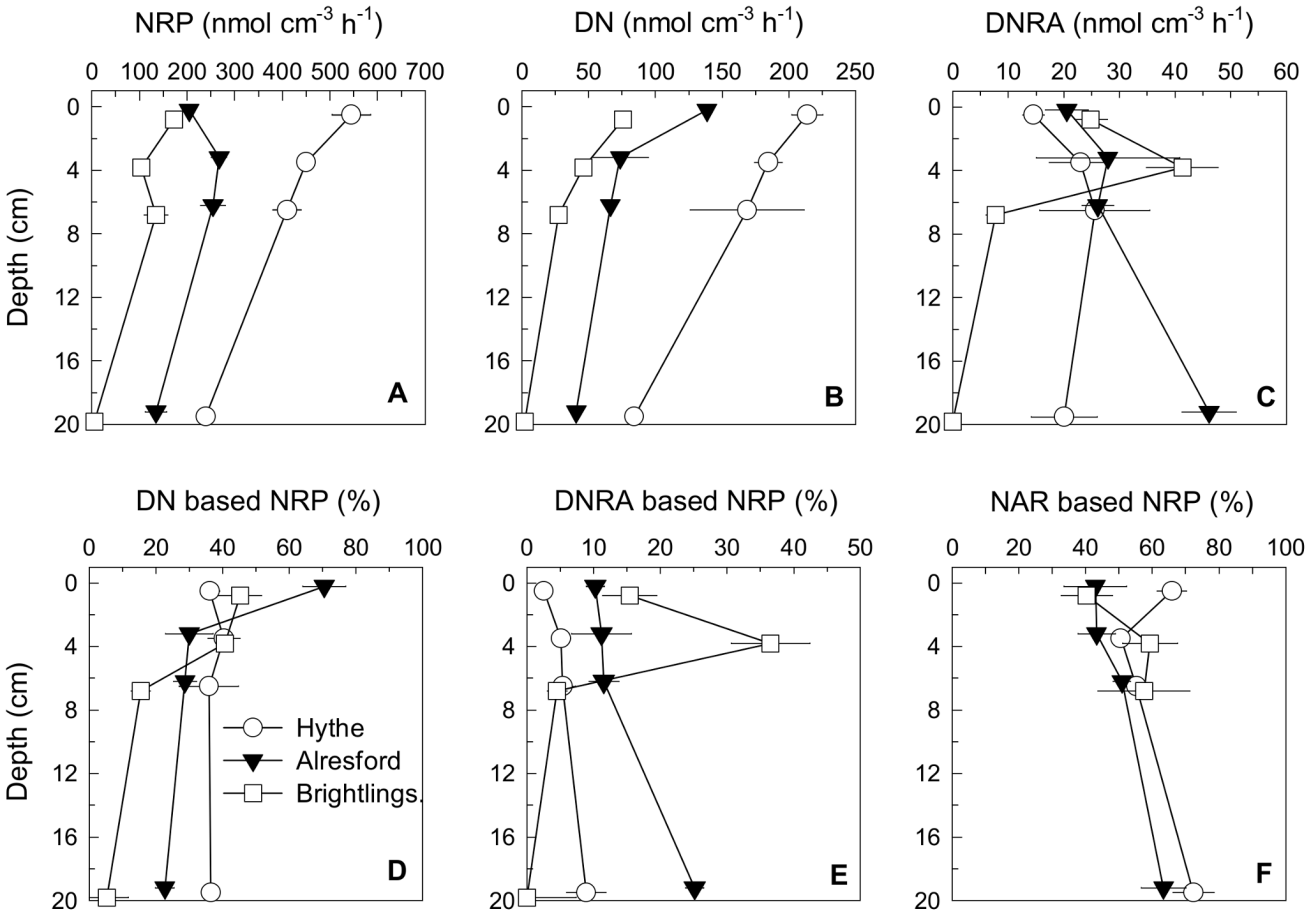
Vertical profiles of sediment nitrate reduction pathways potentials. (A) Nitrate reduction (NRP), (B) denitrification (DN), and (C) dissimilatory nitrate reduction to ammonium (DNRA) potentials, (D) contribution (%) to NRP by DN and (E) by DNRA and (F) contribution (%) of NAR based NRP from slurry experiments conducted with sediment from the Hythe, Alresford and Brightlingsea collected in June 2007. Data points have been offset by 0.2 cm to facilitate observation of error bars. Data are mean ±SE (n = 3).

**Figure 3:**
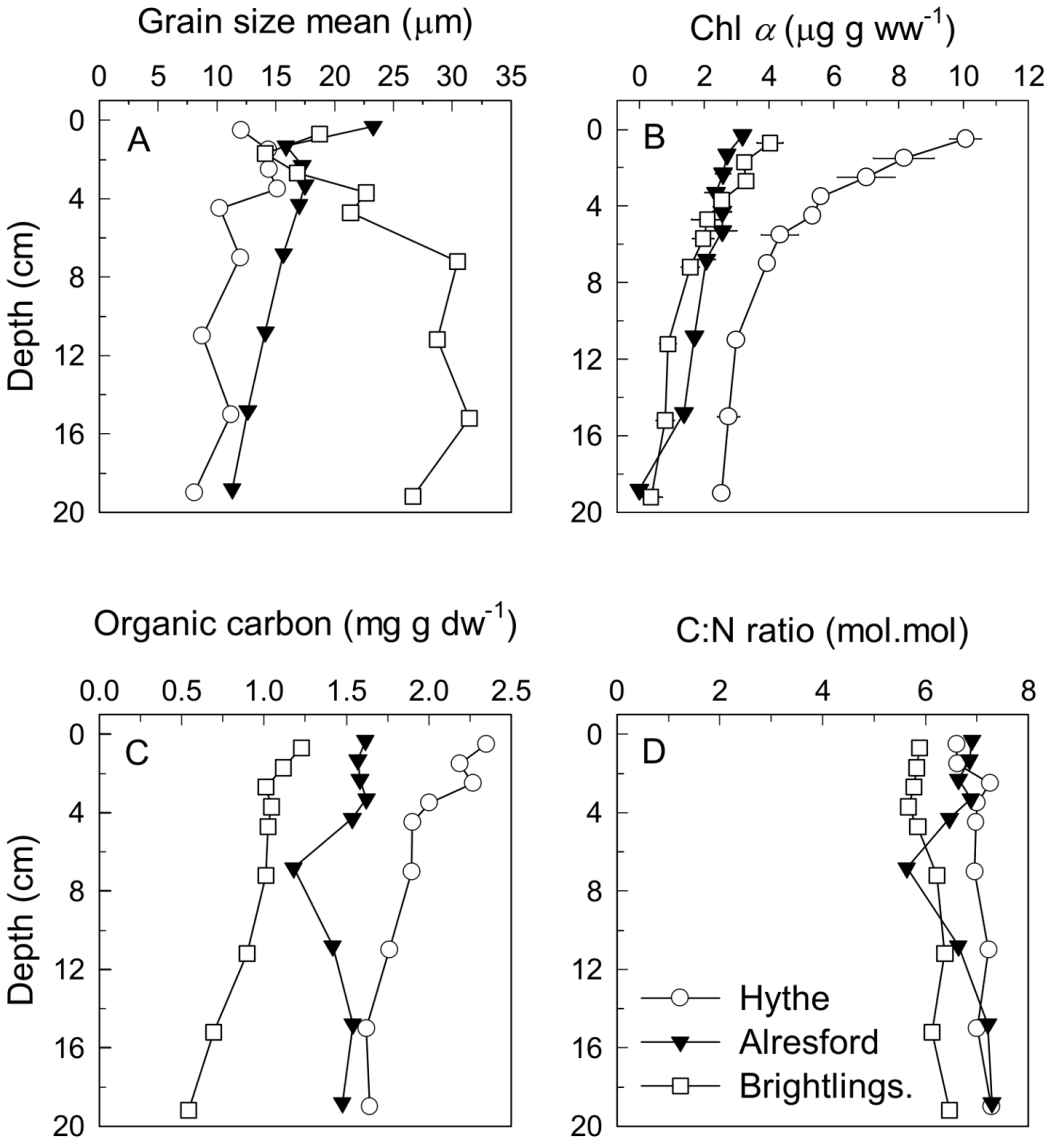
Vertical profiles of sediment nitrate reduction pathways potentials. (A) Nitrate reduction (NRP), (B) denitrification (DN), and (C) dissimilatory nitrate reduction to ammonium (DNRA) potentials, (D) contribution (%) to NRP by DN and (E) by DNRA and (F) contribution (%) of NAR based NRP from slurry experiments conducted with sediment from the Hythe, Alresford and Brightlingsea collected in June 2007. Data points have been offset by 0.2 cm to facilitate observation of error bars. Data are mean ±SE (n = 3).

The measurements of nitrate reduction potentials showed the existence of strong decreasing trends in two dimensions: within each station nitrate reduction potentials were lowest at the deepest layer (*P*<0.001), while at comparable sediment depths the rates decreased significantly from the estuary head to the mouth (*P*<0.001, Table S3) with the exception of the surface sediment at Alresford and Brightlingsea (Fig. 2A). The nitrate reduction potentials observed in the Colne estuary, and especially at the Hythe, are in the upper range of nitrate reduction rates reported from other sediments and soils (Table 3 in [41]) and reflect the high loadings, at least at the Hythe, of C_org_ and N (Fig. 3C, D). Experimental addition of acetate to Hythe slurries significantly increased nitrate reduction potentials rates at all depths (*P*<0.05) (Table S4) showing that, despite the high benthic organic carbon content *in situ* (Fig. 3C), at least for some microorganisms heterotrophic nitrate reduction was simultaneously limited by both electron donor and electron acceptor concentrations. In contrast, at both Alresford and Brightlingsea there was no stimulation by acetate, suggesting that the acetate limited microorganisms were less abundant or absent and that the community between the sites are distinct. Although our results may suggest that nitrate reduction potential rates were solely controlled by nitrate availability at Alresford and Brightlingsea, rates at all three sites could be limited by other organic substrates.

Denitrification potential rates (Fig. 2B) declined from the estuary head (Hythe) to the mouth (Brightlingsea) (P<0.001, Table S3) as nitrate concentrations declined downstream, as shown previously for the Colne and other estuaries [13], [41]–[43], and showing maximum rates near the surface at each site decreasing with depth (*P*<0.001). In contrast, potential DNRA rates increased along the Colne estuary for the first two depths, with the highest rates at the marine site (Fig. 2C). This is in contrast with previously measured *in situ* rates based on ^15^N isotope pairing technique, but agrees with slurry experiments from the Colne performed during the same study [43].

The proportions of nitrate reduced via denitrification or DNRA followed distinct patterns. Assuming that the presence of inhibitors did not change the fates of nitrate, the inhibition of nitrate removal by acetylene suggested approximately 40% of nitrate was denitrified at Hythe (Fig. 2D) without significant differences with depth (P>0.05, Table S3). At Alresford, denitrification accounted for a considerably higher proportion (75%) of the nitrate reduction potential at the sediment surface, but only 25–35% below that depth. Whilst at Brightlingsea, denitrification accounted for 45% in the top two depths, and only 15% at 6–8 cm depth. DNRA potential, on the other hand, increased proportionately from the estuary head to the mouth and from the sediment surface to deeper layers (Fig. 2E). DNRA accounted for 5–10% of nitrate reduction potential at Hythe and 15–25% at Alresford, showing a slight increase with depth, although not statistically significant (P>0.05, Table S3). At Brightlingsea, the highest percentage of DNRA (35%) was at 3–4 cm depth.

Change in the relative significance of denitrification and DNRA has been attributed to changes in the ratio of electron donors to electron acceptors [9], [10], [44]. An increase in the ratio stimulates DNRA relative to denitrification, and in the present case is probably due to a stronger decrease in nitrate concentrations in the water column toward the estuary mouth compared to the concurrent decrease in sediment C_org_ content (Fig. 3C), resulting in lowered donor∶acceptor ratios favouring DNRA. It has been shown that nitrate-ammonifying bacteria are more efficient scavengers of nitrate than denitrifying bacteria [45]. Thus, when competition for nitrate increases down the estuary, reflecting decreasing *in situ* nitrate concentrations, nitrate-ammonifying bacteria might be expected to be competitively more efficient than denitrifying ones. These data would also agree with the % rate data obtained from isotope pairing measurements from the same sites [43].

Denitrification rates showed a significant relationship with the concentration of C_org_ and log transformed functional gene abundance (Tables 1 and 2). However, these relationships vary significantly in their scale (normal-normal, log-normal, log-log), and in their direction depending on the area [43], [46]. Nevertheless, the strong relationship between the variation of the potential denitrification rates and C_org_, C∶N ratio, and log *narG2* and log *nirSe* gene abundance (85%) along the estuary (Table 1) corroborates that these variables play a significant role in the capacity of the sediment to reduce nitrate via denitrification. The same cannot be said for the variation of potential DNRA rates along the estuary, which had only a small relationship (26%) with the environmental or biotic variables. In addition, although it is considered that bacteria capable of performing DNRA would preferentially use nitrate in its presence over other less favourable electron acceptors such as sulphate [47], this might not always be the case [48]. This may explain the lack of expected relationship with variables relevant to DNRA. Therefore, available data so far suggest that most probably some other variables not studied here determine the capacity of the sediment for DNRA in the Colne and that DNRA rates are determined by a more complex array of variables than just denitrification.

**Table 1:** Marginal tests of non-parametric multiple regressions of potential rates.

	Variable	SS trace	pseudo-F	Var (%)
**DN**	Organic carbon	124750.0	105.92***	75.70
	*nirSe*	82543.0	34.12***	50.09
	*nirSm*	80845.0	32.74***	49.06
	*narG2*	67137.0	23.374***	40.74
	C∶N	13616.0	3.06	8.26
	*napA2*	11716.0	2.60	7.11
**DNRA**	*narG2*	1502.80	7.54**	18.16
	Organic carbon	257.66	1.09	3.11
	*napA2*	189.65	0.80	2.29
	C∶N	0.14	0.00	0.00

Potential denitrification (DN) and nitrate reduction to ammonium (DNRA) multiple regressions against environmental and biotic variables for each variable taken individually (ignoring other variables). %Var: percentage of variance in nitrate reduction rate data explained by that variable. There were two groups of highly collinear (r>0.9) variables [*napA1*, *napA3*, *narG1*, *narG2*, *nrfA*] and [*nirSm*, *nirSn*]. Only one variable from each group was included. Functional gene abundances were ln(x+1) transformed. SS: Sums of Squares. Significant relationships are noted with asterisks p<0.05: *, p<0.01 **, p<0.001 ***.

**Table 2:** Overall best solutions of non-parametric multiple regression of potential rates.

	Total SS	AIC	Var (%)	RSS	Variables
**DN**	164790.00	249.16	83.23	27639.00	Organic carbon, C∶N, *narG2, nirSm*
**DNRA**	8275.20	190.96	25.89	6132.40	Organic carbon, *narG2*

The best solution of potential denitrification (DN) and nitrate reduction to ammonium (DNRA) multiple regressions against environmental and biotic variables was found after fitting all possible models and selecting the model with the smallest value of Akaike's Criterion (AIC). %Var: percentage of variance in nitrate reduction rate data explained by the model. There were two groups of highly collinear (r>0.9) variables [*napA1*, *napA3*, *narG1*, *narG2*, *nrfA*] and [*nirSm*, *nirSn*]. Only one variable from each group was included. Functional gene abundances were ln(x+1) transformed. SS: Sums of Squares. RSS: Residual Sum of Squares.

As reported previously [43], only part of the nitrate reduced in the acetylene block experiments with Hythe sediment could be accounted for by the formation of products of denitrification (N_2_O) or DNRA (NH_4_^+^) or of nitrite (between 44%, 0–1 cm, to 58%, 3–4 cm). This value was noticeably higher at Alresford (84% at the surface and 50% for the deeper layers) and Brightlingsea (80% for the two upper layers and 20% for the 6–8 cm layer). It is known that acetylene does not completely inhibit nitrous oxide reductase [49], [50], so we may have underestimated denitrification. Part of the missing reduced nitrate may also be accounted for by Anammox activity as N_2_ formed via Anammox would not have been quantified by the acetylene-inhibited accumulation of N_2_O. Anammox has been suggested to be most important in ecosystems with an excess of N relative to carbon inputs or limited labile carbon [10]. In the Colne, Anammox activity has been estimated to contribute about 30% of N_2_ formation at the Hythe [43] whereas, little or no Anammox activity has been detected at Alresford or Brightlingsea. This agrees with our present finding as the largest missing part of nitrate reduced was in Hythe surface sediments. In addition, nitrite (2–14% of the NO_3_^−^ reduced) only accumulated in the presence of acetylene, a known inhibitor of Anammox [17], at the Hythe but not at the other two sites. Similar observations of highest Anammox activity in the freshwater end of an estuary have been made in Chesapeake Bay [51].

**Figure 4:**
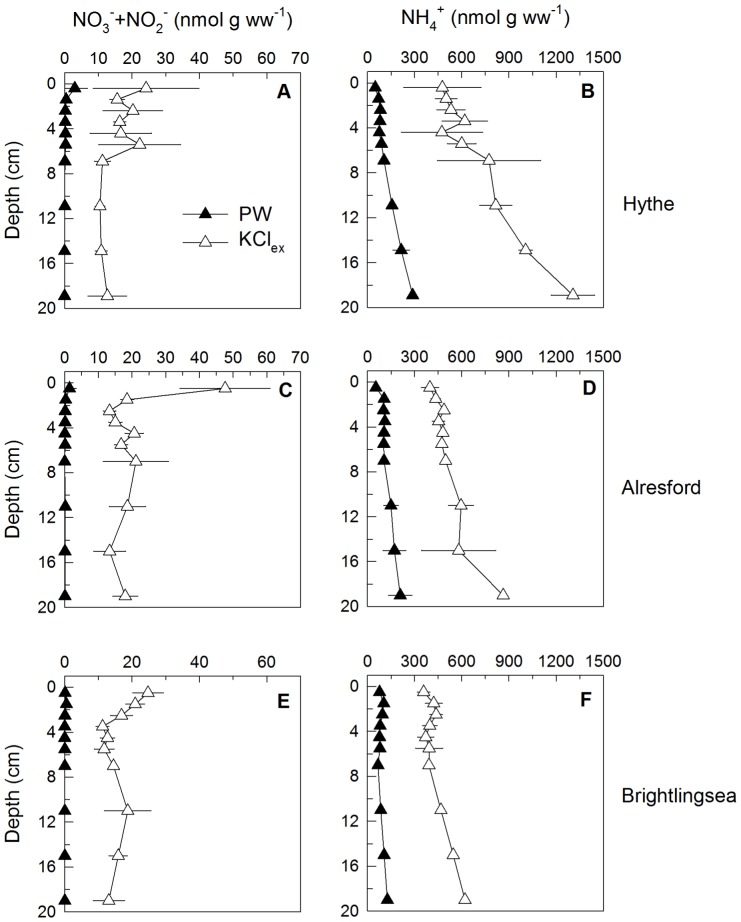
Vertical profiles of sediment nitrate reduction pathways potentials. (A) Nitrate reduction (NRP), (B) denitrification (DN), and (C) dissimilatory nitrate reduction to ammonium (DNRA) potentials, (D) contribution (%) to NRP by DN and (E) by DNRA and (F) contribution (%) of NAR based NRP from slurry experiments conducted with sediment from the Hythe, Alresford and Brightlingsea collected in June 2007. Data points have been offset by 0.2 cm to facilitate observation of error bars. Data are mean ±SE (n = 3).

At the Hythe, C_org_ was 2.5 times higher compared to Brightlingsea although the bulk C∶N ratio, an indication of the quality of organic matter available, was not noticeably different between the three sites with a value of 6–7 (Fig. 3C, 3D). However, the bulk C∶N does not necessarily reflect the C∶N ratio of the available labile sedimentary organic matter pool accessible to bacteria. In addition, porewater nutrients were not different between sites (Fig. 4). At all sites porewater nitrate+ nitrite (NO_x_^−^) was present only in the top 0–1 cm, indicating its rapid consumption within the sediment as it was transported vertically by diffusion from the overlying water (Fig. 4). Therefore, the level of Anammox activity may be high at the Hythe due to very high nitrate concentrations in the overlying water, reaching 1 mM at periods of the year, and where nitrite can also be abundant [12].

### NAP vs NAR contribution to nitrate reduction potential rates

Our results suggested that NAR was proportionately more important than NAP in the surface sediment at the Hythe (NAR 66% of nitrate reduction potential) (Fig. 2F), whereas the opposite was true in Alresford and Brightlingsea (NAR 40–43% of nitrate reduction potential). Richardson [52] argued that periplasmic NAP, which has a higher affinity for nitrate than NAR, is more effective than NAR for nitrate scavenging and subsequent nitrate reduction at low nitrate concentrations and in oxidized environments. This agrees well with the increased importance of NAP at both Alresford and Brightlingsea, where nitrate concentrations are much lower than those at the Hythe [12]. However, at all three sites NAP activity decreased proportionately to NAR with increased sediment depth (NAR being 58–72% of nitrate reduction potential at the deepest depth) (Fig. 2F). This is surprising as an increased importance of NAP would permit the more efficient utilisation of any nitrate that might reach deeper sediments e.g. via bioirrigation.

### Nitrate and nitrite reduction functional genes distribution

**Figure 5:**
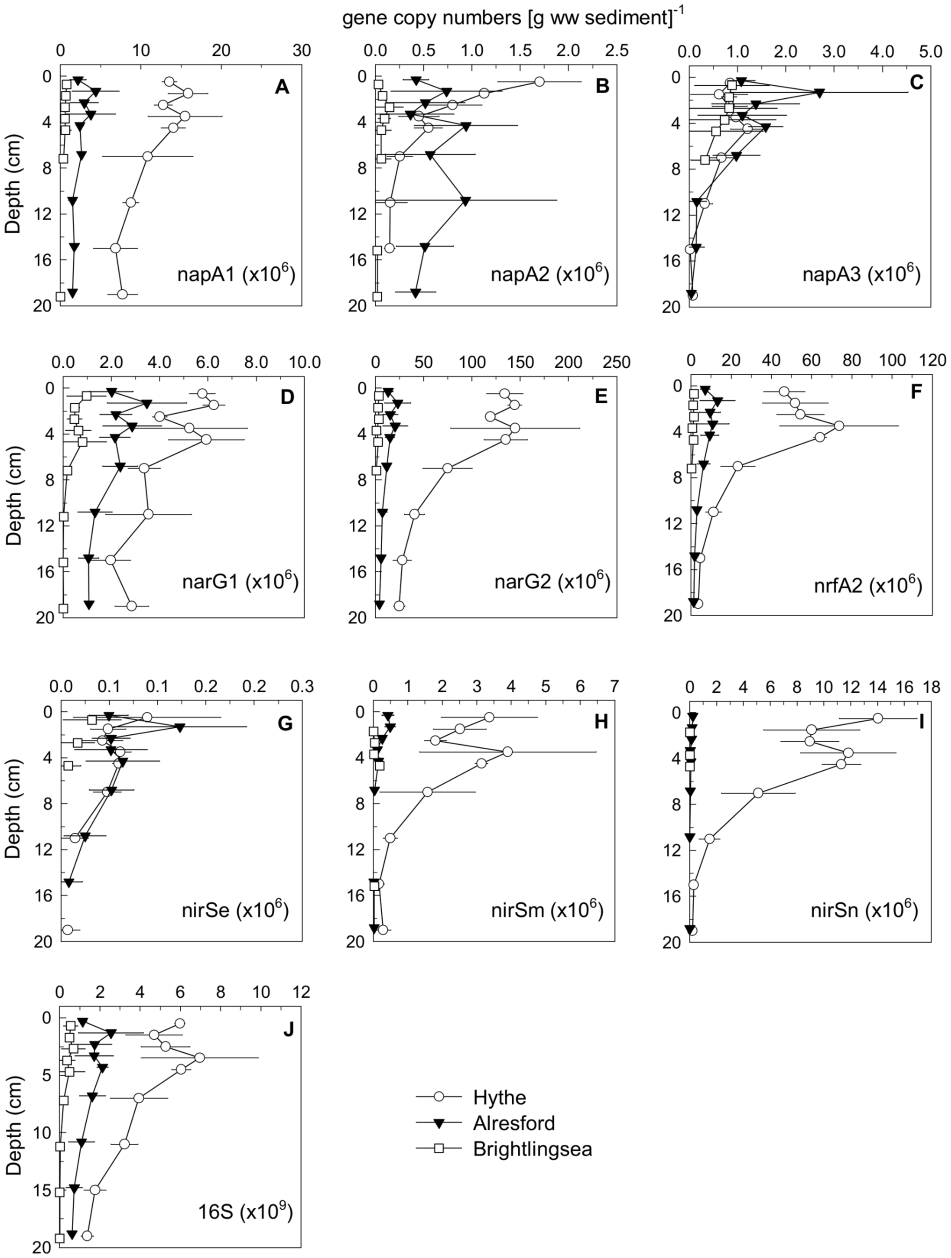
Vertical profiles of sediment 16S rRNA and nitrate reduction functional genes. Abundance of (A) napA1, (B) napA2, (C) napA3, (D) narG1, (E) narG2, (F) nrfA2, (G) nirSe, (H) nirSm, (I) nirSn, and (J) 16S rRNA genes in the sediment at the Hythe, Alresford and Brightlingsea in the Colne estuary in June 2007. Data points have been offset by 0.2 cm to facilitate observation of differences. Missing points are data below detection limit (to distinguish them from low values). Gene copy numbers were calculated from the following standard curves: for napA-1, r^2^ = 0.994,y intercept = 38.74,E(amplification efficiency) = 87.5%, and NTC undetected; for napA-2, r^2^ = 0.992, y intercept = 37.53, E = 85.2%, and NTC undetected; for napA-3, r^2^ = 0.993, y intercept = 40.03, E = 85.5%, and NTC undetected; for narG-1, r^2^ = 0.999, y intercept = 39.40, E = 92.3%, and NTC undetected; for narG-2, r^2^ = 0.998, y intercept = 41.14, E = 84.8%, and NTC undetected; for nrfA-2, r^2^ = 0.999, y intercept = 42.13, E = 85.8%, and NTC undetected; for nirS-e, r^2^ = 0.998, y intercept = 39.06, E = 88.7%, and NTC undetected; for nirS-m, r^2^ = 0.996, y intercept = 38.37, E = 86.6%, and NTC undetected; for nirS-n, r^2^ = 0.995, y intercept = 39.38, E = 89.3%, and NTC undetected; and for 16S rDNA, r^2^ = 0.996, y intercept = 40.96, E = 86.2%, and Ct cutoff = 34.98.

Although there were some variations with depth and among different phylotypes, overall there were significant decreases in 16S rRNA and functional gene copy numbers (*P*<0.05, Table S5) of the most abundant phylotypes of *narG*, *napA*, *nirS* and *nrfA* genes from the Hythe to Brightlingsea and from the surface sediments to deeper layers (Fig. 5). In contrast, two of the three *napA* phylotypes (*napA2* and *napA3*) and one of the *nirS* (*nirSe*) did not show significant differences in numbers between the three sites along the estuary, which is in agreement with previous studies [14], [43]. Consistent trends in gene copy numbers can be observed between the different studies for surface sediments along the Colne estuary indicating that the patterns between sites remain, but within site temporal variations occur in the numbers of the nitrate- and nitrite- reducing bacteria.

Various environmental variables (e.g., NO_3_^−^,NO_2_^−^,NH_4_^+^, O_2_, salinity) have been suggested to affect the composition and distribution of the nitrate reducing communities in marine sediments [46], [53]–[55]. Examination of the relationships between the distribution of the genes assemblages and the sediment environmental variables revealed that sediment grain size (38.0%), C_org_ (37%), and chlorophyll *a* (20%) were significant in explaining the distribution of the functional gene assemblages along the estuary and with depth (Tables 3 and 4). Although the variables selected by such an analysis should not be interpreted as being necessarily causative, it is a strong suggestion that these factors may have an effect on the distribution of the relevant bacterial populations. However, it is clear that the assemblages on the whole change considerably along the estuary and that these changes are more evident for the surface rather than deeper sediments.

**Table 3:** Non-parametric multiple regression marginal tests of multivariate nitrate reduction functional gene data.

Variable	SS trace	pseudo-F	Var (%)
**Grain size**	6688.0	15.55***	38.4
**Organic carbon**	6510.1	14.89***	37.3
**Chlorophyll ** ***a***	3467.1	6.20**	19.9
**Porewater NH_4_^+^**	1746.7	2.78	10.0
**C∶N**	1547.6	2.43	8.9
**Porewater NO_x_-**	832.3	1.25	4.8
**KCl_ex_.NH_4_^+^**	495.1	0.73	2.8
**KCl_ex_ NO_x_-**	367.0	0.53	2.1

Sediment environmental variables were tested individually (ignoring other variables) %Var: percentage of variance in nitrate reduction functional gene abundance data explained by that variable. KCl_ex_: Freeze lysable plus KCl extractable pool. SS: Sums of Squares. Significant relationships are noted with asterisks p<0.05: *, p<0.01 **, p<0.001 ***.

**Table 4:** Overall best models of non-parametric multiple regression of multivariate nitrate reduction functional gene data.

AIC	Var (%)	RSS	Variables
**160.14**	57.91	7339.6	Grain size, Organic carbon, KCl_ex_.NH_4_^+^
**160.81**	55.56	7749.3	Grain size, Porewater NH_4_^+^, KCl_ex_.NH_4_^+^
**161.23**	58.08	7309.6	Grain size, Organic carbon, KCl_ex_.NH_4_^+^, KCl_ex_ NO_x_-

The three best overall solutions were determined after fitting all of the possible combinations of models and selecting the ones with the smallest value of Akaike's Criterion (AIC). %Var: percentage of variance in nitrate reduction functional gene abundance data explained by the model. RSS: Residual Sum of Squares. KCl_ex_: Freeze lysable plus KCl extractable pool.

### Nitrate reduction deeper in the sediment. Why?

The vertical profiles of 16S rRNA and key functional gene copy numbers showed the highest values near the top 4 cm at the Hythe, below which they declined (Fig. 5); reflecting the decrease in nitrate reduction potential with increased depth. The presence of a functional gene does not mean that it is actually active *in situ* and in many cases there is significant disagreement between gene copy and/or transcript abundance and rate processes (i.e. activity) [43], [56], although generally functional gene abundance reflect recent process activity and show good correlation with potential rates [43], [46], [57]. It is still surprising though why measurable nitrate reduction potential, denitrification rates, or nitrate reduction pathway functional genes, are found in deeper sediments, which are unlikely to be exposed to nitrate in the porewater [41], [55], [58], [59]. In usually resource-limited and relatively constant natural environments, gene loss of dispensable functions can provide a selective advantage by conserving an organism's limiting resources [60], [61]. Why then are nitrate reduction genes and the capacity for nitrate reduction maintained within these deeper sediments? Introduction of nitrate by advection is unlikely since the sediments consisted mainly of fine to coarse silt (Fig. 3A) and are well consolidated with surface microalgal biofilms [13], [62]. The transport of nitrate to deeper sediment layers by bioirrigation, with its rapid removal from the porewater, is one possibility to explain the maintenance of nitrate reduction capacity. Indeed, an abundant bioturbating infauna was found at the Hythe, comprising mainly of the polychaete *Nereis diversicolor* (2500 ind. m^−2^), the amphipod *Corophium* sp. (1000 ind. m^−2^) and capitellid polychates (30000 ind. m^−2^). The abundance of these groups was lower at Alresford; in contrast, showing greater abundance of molluscs (1800 ind. m^−2^). At Brightlingsea, the community showed lower abundances overall and was characterised primarily by the presence of *Nepthys* sp (400 ind. m^−2^), spionids (2000 ind.m^−2^) and capitellids (5000 ind. m^−2^). Transport of nitrate through *Nereis diversicolor* burrows could stimulate DN but usually this occurs only down to 10 cm depth [63], [64]. In fact, porewater NH_4_^+^ showed the typical profile of well-mixed bioturbated sediment in the upper 8 cm, increasing with depth below this (Fig. 4).

Many sulphate reducers also have the capability of nitrate reduction when nitrate is available [47], as in our slurry experiments, although *in situ* in the absence of nitrate any adaptive advantage would be negligible. However, sulphate reducing bacteria perform DNRA and not denitrification. Indeed, some of the Colne *nrfA* phylotypes have been related to sulphate reducers [14], [65] and *nrfA2* copy numbers in our study peaked at 3–5 cm depth (Fig. 5), concurrent with the depth where sulfate reduction tends to be highest in the Colne [66]. Although this could explain DNRA in deeper sediments, it does not account for the detection of potential denitrification at depth. Furthermore, the nitrate reducing community assemblage was different between surface and deeper sediments. While some phylotypes of the genes studied decreased almost exponentially with depth, others were less variable with depth (Fig. 5). Despite differences often found between a gene's abundance and levels of expression, as mentioned previously, the differences in the vertical pattern of the various phylotypes reasonably suggests differences in their activity throughout the sediment column. This raises interesting questions as to what the alternative metabolic roles for the various nitrate reductases could be and why some are not selected against in the deeper sediments where the lack of porewater nitrate renders them redundant. Given that the gene sequences isolated from these systems are novel in comparison with the same genes from cultured isolates [14], [15], it may be possible that the environmental sequences have different functionalities as proteins. In fact, some nitrite reductases are optimized for the reduction of different substrates (e.g. sulphite, nitric oxide, hydroxylamine) in different organisms and perform apart from respiratory nitrite ammonification also nitrogen compound detoxification and respiratory sulfite reduction [67], [68]. If this is the case, then that could be a possible explanation for the disconnect between gene presence and *in situ* biogeochemistry.

The pattern of freeze-lysable KCl-extractable (KCl_ex_) nutrients followed that of porewater nutrients; a decrease with depth for NO_x_^−^ and an increase for NH_4_^+^, albeit at much higher concentrations. While KCl_ex_ NH_4_^+^ was about 5-fold the porewater concentration, KCl_ex_ NO_x_^−^ was on average about 300-fold higher than that of its porewater concentration (Fig. 4). One source of these high NO_x_^−^ concentrations could be intracellular pools; cell rupture by freezing and KCl extraction can release NO_x_^−^ from high concentration intracellular pools, as shown elsewhere [69], [70]. Active chlorophyll was detected even down to 20 cm depth (Fig. 3B), suggesting vertical migration or transport of microbenthic algae which are effective scavengers of nitrate [71], [72] and while intracellular pools of nitrate in most algal cells are not particularly high, Garcia-Robledo et al [70] showed a correlation between benthic microalgae and pools of freeze-lysable nitrate at least for near surface sediments. Risgaard-Petersen et al. [73], on the other hand, showed very high intracellular nitrate pools in foraminifera, which can be abundant in sediments and which are capable of denitrification [73], [74]. However, the most likely candidates for the high NO_x_^−^ concentrations and the nitrate reducing genes would be facultative sulphide oxidisers such as *Thioploca* or sulfur/sulfide oxidizing *Beggiatoa* spp. These bacteria accumulate nitrate in their cytoplasm to very high concentrations (∼500–1000 mM) [75] in the oxic layers of sediment before migrating down into anoxic, high sulphide sediments where the nitrate is used as an electron acceptor. Therefore, microalgal, foraminiferal or *Thioploca/Beggiatoa*-type organisms could be responsible for the presence of high levels of KCl_ex_ nitrate and key nitrate reduction genes in the anoxic sediment profile.

To determine whether the presence of nitrate reduction genes in deeper sediments (where porewater nitrate was absent) was due to these nitrate-accumulating bacteria in the sediment, pyrosequencing was performed. With this pyrosequencing analysis, our main aim was to identify if nitrate-accumulating bacteria were present at high abundance within the sediment samples and thus likely to be having significant influence on our functional (nutrient) data. Out of a total of 70,979 (remaining sequences after quality checking) 16S rRNA gene sequences recovered from the Colne, none were specific for *Thioploca* (Table S6). This was confirmed by using both the RDP classifier algorithm matching our pyrosequencing data against a comprehensive reference collection of 16S rRNA sequences and via pairwise Needleman-Wunsch alignments of known *Thioploca* spp. sequences against all our pyrosequence reads. However, two sequences relating to *Cycloclasticus* spp (a closely related species) were recovered from the upper sediments at Brightlingsea, which confirmed that the primers used were able to identify members of the Thiotrichales, if present. However, it must be noted that our sequencing intensity was not extensive (i.e. non-asymptotically sampled rarefaction curves); subsequently a large portion of estuarine sediment biodiversity may have been overlooked. Yet, microbial taxa in high enough abundance to influence the nitrate-reduction processes we measured would likely have been detected. Thus, it is parsimonious to consider that the general absence of these sequences in the libraries indicates that *Thioploca/Beggiatoa* are not responsible in the Colne for the subsurface presence of either the KCl_ex_ NO_x_^−^ or the functional genes for denitrification, but that we must hypothesise other bacteria, microalgae or foraminifera as their source. Although our data does not allow us to distinguish between the intracellular and easily exchangeable pools, the role of exchangeable nitrate in estuarine sediments [76], [77] and the degree of bioavailability of this exchangeable pool still remains to be examined.

## Supporting Information

Table S1 **Primer and probe sets.** Primer and probe sets used for DNA extraction efficiency tests, pyrosequencing analysis, and *qPCR* of functional nitrate reduction genes.
(DOCX)

Table S2 **Michaelis-Menten rate expression statistics on potential nitrate reduction rates.** Statistical analysis results obtained by fitting a Michaelis-Menten rate expression on potential nitrate reduction rates from the Hythe, Alresford and Brightlingsea of the Colne estuary collected in May 2007. No curve could be calculated for the bottom layer at Brightlingsea.
(DOCX)

Table S3 **PERMANOVA results of data from potential rates experiments.** PERMANOVA results on measured NRR, DN and DNRA rates, and % contribution of DN and nar based nitrate reduction in sediment slurries at different depths (factor Depth) along the Colne estuary (factor Site). Homogeneous groups from post hoc analysis are shown with superscript letters at a p<0.05 level. Ns: non significant differences. H: Hythe, A: Alresford, B: Brightlingsea. 0: 0–1 cm, 3: 3–4 cm, 6: 6–8 cm, 18:18–20 cm.
(DOCX)

Table S4 **PERMANOVA results of acetate addition effect on nitrate reduction rates.** PERMANOVA analysis of nitrate reduction rates (nmol cm^−3^ h^−1^) in slurry experiments without and with added acetate (10 mM) (factor Acetate) at different sediment depths (factor Depth) along the Colne estuary.
(DOCX)

Table S5 **PERMANOVA results of functional genes abundance in the Colne.** PERMANOVA table for measured functional genes abundance at different depths (factor Depth) along the Colne estuary (factor Site). Homogeneous groups from post hoc analysis are shown with superscript letters at a p<0.05 level. Ns: non significant differences. H: Hythe, A: Alresford, B: Brightlingsea. Numbers (0, 1, 2, 3, 4, 6, 10, 14 and 18) represent upper limit of depth layers. Depth layers that show similar patterns are grouped together and underlined.
(DOCX)

Table S6 **Pyrosequencing analysis results.** Total number of sequences and percentage of sequences at different depths along the Colne estuary in June 2007. H: Hythe, A: Alresford, B: Brightlingsea. Numbers (0, 1, 2, 3, 4, 6, 10, 14 and 18) represent upper limit of depth layers. Values in bold represent contributions above 1% of the sequences in the sample.
(DOCX)
