# Supplementary material for: Nitrate Reduction Functional Genes and Nitrate Reduction Potentials Persist in Deeper Estuarine Sediments. Why?
Source: PLoS One. 2014 Apr 11;9(4):e94111. doi: 10.1371/journal.pone.0094111 (PMC3984109; doi:10.1371/journal.pone.0094111)
Supplement: Table S1 — Primer and probe sets. Primer and probe sets used for DNA extraction efficiency tests, pyrosequencing analysis, and qPCR of functional nitrate reduction genes. (DOCX) [file pone.0094111.s001.docx]

**Table S1**. **Primer and probe sets**. Primer and probe sets used for DNA extraction efficiency tests, pyrosequencing analysis, and *qPCR* of functional nitrate reduction genes.

| Analysis | Target gene | Phylotype | Primer/probe name ^a^ | Primer or probe sequence  (5′ - 3′) | Reference |
| --- | --- | --- | --- | --- | --- |
| *DNA extraction testing* | *16S* |  | 1369F | CGG TGA ATA CGT TCY CGG | [[1](#_ENREF_1)] |
|  |  |  | 1492R | GGW TAC CTT GTT ACG ACT T |  |
| *Pyrosequencing* | *16S* |  | Gray28F | GAGTTTGATCNTGGCTCAG . | [[2](#_ENREF_2)] |
|  |  |  | Gray519R | GTNTTACNGCGGCKGCTG |  |
| *qPCR* | *napA* | napA-1 | napA-1F | GTY ATG GAR GAA AAA TTC AA | [[3](#_ENREF_3)] |
|  |  |  | napA-1R | GAR CCG AAC ATG CCR AC |  |
|  |  |  | napA-1 TM MGB probe | AAC ATG ACC TGG AAG |  |
| *qPCR* | *napA* | napA-2 | napA-2F | GAA CCK AYG GGY TGT TATG | [[3](#_ENREF_3)] |
|  |  |  | napA-2R | TGC ATY TCS GCC ATR TT |  |
|  |  |  | napA-2 TM MGB probe | CTT TGG GGT TCA A |  |
| *qPCR* | *napA* | napA-3 | napA-3F | CCC AAT GCT CGC CAC TG | [[3](#_ENREF_3)] |
|  |  |  | napA-3R | CAT GTT KGA GCC CCA CAG |  |
|  |  |  | napA-3 TM MGB probe | TGG GTT GTT ACG A |  |
| *qPCR* | *narG* | narG-1 | narG-1F | GAC TTC CGC ATG TCR AC | [[3](#_ENREF_3)] |
|  |  |  | narG-1R | TTY TCG TAC CAG GTG GC |  |
|  |  |  | narG-1 TM MGB probe | TAY TCC GAC ATC GT |  |
| *qPCR* | *narG* | narG-2 | narG-2F | CTC GAY CTG GTG GTY GA | [[3](#_ENREF_3)] |
|  |  |  | narG-2R | TTY TCG TAC CAG GTS GC |  |
|  |  |  | narG-2 TM MGB probe | AAC TTC CGC ATG GA |  |
| *qPCR* | *nrfA* | nrfA-2 | nrfA-2F | CAC GAC AGC AAG ACT GCC G | [[3](#_ENREF_3)] |
|  |  |  | nrfA-2R | CCG GCA CTT TCG AGC CC |  |
|  |  |  | nrfA TM MGB probe | TTG ACC GTC GGC A |  |
| *qPCR* | *nirS* | nirS-e | nirS-eF | CAC CCG GAG TTC ATC GTC | [[3](#_ENREF_3)] |
|  |  |  | nirS-eF | ACC TTG TTG GAC TGG TGG G |  |
|  |  |  | nirS-e TM MGB probe | TGC TGG TCA ACT A |  |
|  |  | nirS-m | nirS-mF | GGA AAC CTG TTC GTC AAG AC | [[3](#_ENREF_3)] |
|  |  |  | nirS-mR | CSG ART CCT TGG CGA CGT |  |
|  |  |  | nirS-m TM probe | TCT GGG CCG ACG CGC CGA TGA AC |  |
|  |  | nirS-n | nirS-nF | AAG GAA GTC TGG ATY TC | [[3](#_ENREF_3)] |
|  |  |  | nirS-nR^b^ | CGT TGA ACT TRC CGG T |  |
|  |  |  | nirS-n TM MGB probe | ATC CGA AGA TSA |  |

^a^ For probes: TM-MGB, TaqMan minor groove binding; TM, TaqMan.

^b^ Also known as nirS6r [[4](#_ENREF_4)].

**References**

1. Suzuki MT, Taylor LT, DeLong EF (2000) Quantitative analysis of small-subunit rRNA genes in mixed microbial populations via 5 '-nuclease assays. Appl Environ Microbiol 66: 4605-4614.

2. Ishak HD, Plowes R, Sen R, Kellner E, Meyer E, et al. (2011) Bacterial diversity in *Solenopsis invicta* and *Solenopsis geminata* ant colonies characterized by 16S amplicon 454 pyrosequencing. Microb Ecol 61: 821-831.

3. Smith CJ, Nedwell DB, Dong LF, Osborn AM (2007) Diversity and abundance of nitrate reductase genes (narG and napA), nitrite reductase genes (nirS and nrfA), and their transcripts in estuarine sediments. Appl Environ Microbiol 73: 3612-3622.

4. Braker G, Fesefeldt A, Witzel KP (1998) Development of PCR primer systems for amplification of nitrite reductase genes (*nirK* and *nirS*) to detect denitrifying bacteria in environmental samples. Appl Environ Microbiol 64: 3769-3775.
