# Supplementary material for: Nitrate Reduction Functional Genes and Nitrate Reduction Potentials Persist in Deeper Estuarine Sediments. Why?
Source: PLoS One. 2014 Apr 11;9(4):e94111. doi: 10.1371/journal.pone.0094111 (PMC3984109; doi:10.1371/journal.pone.0094111)
Supplement: Table S2 — Michaelis-Menten rate expression statistics on potential nitrate reduction rates. Statistical analysis results obtained by fitting a Michaelis-Menten rate expression on potential nitrate reduction rates from the Hythe, Alresford and Brightlingsea of the Colne estuary collected in May 2007. No curve could be calculated for the bottom layer at Brightlingsea. (DOCX) [file pone.0094111.s002.docx]

**Table S2**. **Michaelis-Menten rate expression statistics on potential nitrate reduction rates**. Statistical analysis results obtained by fitting a Michaelis-Menten rate expression on potential nitrate reduction rates from the Hythe, Alresford and Brightlingsea of the Colne estuary collected in May 2007. No curve could be calculated for the bottom layer at Brightlingsea.

| Site | Depth (cm) | R^2^ | F | p |
| --- | --- | --- | --- | --- |
| Hythe | 0-1 | 0.989 | 361.519 | <0.0001 |
|  | 3-4 | 0.959 | 94.525 | 0.0006 |
|  | 6-8 | 0.901 | 36.244 | 0.0038 |
|  | 18-20 | 0.879 | 28.956 | 0.0058 |
| Alresford | 0-1 | 0.945 | 69.168 | 0.0011 |
|  | 3-4 | 0.936 | 58.885 | 0.0016 |
|  | 6-8 | 0.906 | 38.507 | 0.0034 |
|  | 18-20 | 0.941 | 48.072 | 0.0062 |
| Brightlingsea | 0-1 | 0.988 | 321.699 | <0.0001 |
|  | 3-4 | 0.802 | 16.225 | 0.0158 |
|  | 6-8 | 0.862 | 24.958 | 0.0075 |
|  | 18-20 | - | - | - |
