# Supplementary material for: Nitrate Reduction Functional Genes and Nitrate Reduction Potentials Persist in Deeper Estuarine Sediments. Why?
Source: PLoS One. 2014 Apr 11;9(4):e94111. doi: 10.1371/journal.pone.0094111 (PMC3984109; doi:10.1371/journal.pone.0094111)
Supplement: Table S3 — PERMANOVA results of data from potential rates experiments. PERMANOVA results on measured NRR, DN and DNRA rates, and % contribution of DN and nar based nitrate reduction in sediment slurries at different depths (factor Depth) along the Colne estuary (factor Site). Homogeneous groups from post hoc analysis are shown with superscript letters at a p<0.05 level. Ns: non significant differences. H: Hythe, A: Alresford, B: Brightlingsea. 0: 0–1 cm, 3: 3–4 cm, 6: 6–8 cm, 18:18–20 cm. (DOCX) [file pone.0094111.s003.docx]

**Table S3**. **PERMANOVA results of data from potential rates experiments**. PERMANOVA results on measured NRR, DN and DNRA rates, and % contribution of DN and nar based nitrate reduction in sediment slurries at different depths (factor Depth) along the Colne estuary (factor Site). Homogeneous groups from post hoc analysis are shown with superscript letters at a p<0.05 level. Ns: non significant differences. H: Hythe, A: Alresford, B: Brightlingsea. 0: 0-1 cm, 3: 3-4 cm, 6: 6-8 cm, 18:18-20 cm.

|  | Source | df | MS_resid_ | Pseudo-F | p |  | Post hoc tests | | | |
| --- | --- | --- | --- | --- | --- | --- | --- | --- | --- | --- |
| NRR | Site | 2 | 2689.3 | 214.33 | 0.0001 |  | **H**: 0^a^, 3^ab^, 6^b^, 18^c^ | **A**: 0^a^, 3^b^, 6^ab^, 18^c^ | **B**: 0^a^, 3^b^, 6^a,b^, 18^c^ |  |
|  | Depth | 3 |  | 42.162 | 0.0001 |  | **0**: H^a^, A^b^, B^b^ | **3**: H^a^, A^b^, B^c^ | **6**: H^a^, A^b^, B^c^ | **18**: H^a^, A^b^, B^c^ |
|  | SxD | 6 |  | 6.5623 | 0.0002 |  |  |  |  |  |
| DN | S | 2 | 646.36 | 75.081 | 0.0001 |  | H^a^, A^b^, B^c^ |  |  |  |
|  | D | 3 |  | 23.859 | 0.0001 |  | 0^a^, 3^b^, 7^b^, 18^c^ |  |  |  |
|  | SxD | 6 |  | 1.5361 | 0.2114 |  |  |  |  |  |
| DNRA | S | 2 | 105.33 | 5.2048 | 0.014 |  | **H**: 0^a^, 3^a^, 7^b^, 18^b^ | **A**: 0^a^, 3^ab^, 6^a^, 18^b^ | **B**: 0^a^, 3^b^, 6^c^, 18^d^ |  |
|  | D | 3 |  | 2.3222 | 0.1006 |  | **0**: H^a^, A^ab^, B^b^ | **3**: H^a^, A^a^, B^a^ | **6**: H^ab^, A^a^, B^b^ | **18**: H^a^, A^b^, B^c^ |
|  | SxD | 6 |  | 5.4457 | 0.001 |  |  |  |  |  |
| %DN | S | 2 | 48.952 | 7.0017 | 0.0047 |  | **H**: ns | **A**: 0^a^, 3^b^, 6^b^, 18^ab^ | **B**: 0^a^,3^a^, 6^b^, 18^b^ |  |
|  | D | 3 |  | 10.95 | 0.0003 |  | **0**: H^a^, A^b^, B^ab^ | **3**: n.s | **6**: ns | **18**: H^a^, A^ab^, B^b^ |
|  | SxD | 6 |  | 5.0234 | 0.0022 |  |  |  |  |  |
| %DNRA | S | 2 | 31.371 | 11.47 | 11.47 |  | **H**: ns | **A**: ns | **B**: 0^a^,3^b^, 6^c^, 18^c^ |  |
|  | D | 3 |  | 3.5307 | 3.5307 |  | **0**: H^a^, A^b^, B^b^ | **3**: H^a^, A^a^, B^b^ | **6**: ns | **18**: H^ab^, A^a^, B^b^ |
|  | SxD | 6 |  | 12.061 | 12.061 |  |  |  |  |  |
| %nar | S | 2 | 56.353 | 10.354 | 0.0009 |  | **H**: 0^ab^, 3^a^, 6^a^, 18^b^ | **A**: ns | **B**: 0^a^, 3^ab^, 6^b^, 18^c^ |  |
|  | D | 3 |  | 4.396 | 0.0123 |  | **0**: ns | **3**: ns | **6**: H^a^, A^a^, B^b^ | **18**: H^a^, A^a^, B^b^ |
|  | SxD | 6 |  | 19.8 | 0.0001 |  |  |  |  |  |
