# Supplementary material for: Nitrate Reduction Functional Genes and Nitrate Reduction Potentials Persist in Deeper Estuarine Sediments. Why?
Source: PLoS One. 2014 Apr 11;9(4):e94111. doi: 10.1371/journal.pone.0094111 (PMC3984109; doi:10.1371/journal.pone.0094111)
Supplement: Table S4 — PERMANOVA results of acetate addition effect on nitrate reduction rates. PERMANOVA analysis of nitrate reduction rates (nmol cm−3 h−1) in slurry experiments without and with added acetate (10 mM) (factor Acetate) at different sediment depths (factor Depth) along the Colne estuary. (DOCX) [file pone.0094111.s004.docx]

**Table S4**. **PERMANOVA results of acetate addition effect on nitrate reduction rates**. PERMANOVA analysis of nitrate reduction rates (nmol cm^-3^ h^-1^) in slurry experiments without and with added acetate (10 mM) (factor Acetate) at different sediment depths (factor Depth) along the Colne estuary.

|  | Source | df | MS_res_ | Pseudo-F | P(MC) |
| --- | --- | --- | --- | --- | --- |
| Hythe | Acetate | 1 | 7941.8 | 7.9583 | **0.0118** |
|  | Depth | 3 |  | 82.647 | **0.0001** |
|  | AcxD | 3 |  | 0.9364 | 0.4474 |
| Alresford | Acetate | 1 | 2922 | 1.6254 | 0.2255 |
|  | Depth | 3 |  | 6.5583 | **0.0048** |
|  | AcxD | 3 |  | 1.061 | 0.399 |
| Brightlingsea | Acetate | 1 | 1704.4 | 0.39286 | 0.5424 |
|  | Depth | 3 |  | 15.052 | **0.0006** |
|  | AcxD | 3 |  | 0.19643 | 0.9026 |
