# Supplementary material for: Nitrate Reduction Functional Genes and Nitrate Reduction Potentials Persist in Deeper Estuarine Sediments. Why?
Source: PLoS One. 2014 Apr 11;9(4):e94111. doi: 10.1371/journal.pone.0094111 (PMC3984109; doi:10.1371/journal.pone.0094111)
Supplement: Table S5 — PERMANOVA results of functional genes abundance in the Colne. PERMANOVA table for measured functional genes abundance at different depths (factor Depth) along the Colne estuary (factor Site). Homogeneous groups from post hoc analysis are shown with superscript letters at a p<0.05 level. Ns: non significant differences. H: Hythe, A: Alresford, B: Brightlingsea. Numbers (0, 1, 2, 3, 4, 6, 10, 14 and 18) represent upper limit of depth layers. Depth layers that show similar patterns are grouped together and underlined. (DOCX) [file pone.0094111.s005.docx]

**Table S5.** **PERMANOVA results of functional genes abundance in the Colne**. PERMANOVA table for measured functional genes abundance at different depths (factor Depth) along the Colne estuary (factor Site). Homogeneous groups from post hoc analysis are shown with superscript letters at a p<0.05 level. Ns: non significant differences. H: Hythe, A: Alresford, B: Brightlingsea. Numbers (0, 1, 2, 3, 4, 6, 10, 14 and 18) represent upper limit of depth layers. Depth layers that show similar patterns are grouped together and underlined.

|  | Source | df | MSres | Pseudo-F | perms | p | Post hoc tests |
| --- | --- | --- | --- | --- | --- | --- | --- |
| *napA1* | St | 2 | 1.3834 | 135.21 | 280 | 0.0001 | **3**: H^a^, A^ab^, B^b^; **0,1, 4**: H^a^, A^b^, B^b^; **2, 6,10,14,18**: H^a^, A^b^, B^c^ |
|  | De | 8 |  | 31.709 | 9946 | 0.0001 | **H**: 0^ab^ 1^ab^ 2^ab^ 3^ab^ 4^a^ 6^a^ 10^b^ 14^b^ 18^ab^; **A**: ns; **B**:0^a^ 1^a^ 2^a^ 3^ab^ 4^ab^ 6^a^ 10^b^ 14^b^ 18^ab^ |
|  | Co(St) | 6 |  | 2.0048 | 9949 | 0.08 |  |
|  | StxDe | 16 |  | 24.283 | 9930 | 0.0001 |  |
|  |  |  |  |  |  |  |  |
| *napA2* | St | 2 | 6.7093 | 53.746 | 279 | 0.0037 | **0**: H^a^, A^b^, B^c^; **1**: H^a^, A^ab^, B^b^; **2,3,4,6,15**:ns ; **10**: H^a^, A^a^, B^b^; **18**: H^a^, A^b^, B^ab^ |
|  | De | 8 |  | 6.1153 | 9936 | 0.0001 | **H**: 0^a^ 1^a^ 2^a^ 3^a^ 4^a^ 6^ab^ 10^ab^ 14^ab^ 18^b^; **A**: ns; **B**:0^ab^ 1^a^ 2^ab^ 3^ab^ 4^ab^ 6^ab^ 10^b^ 14^ab^ 18^ab^ |
|  | Co(St) | 6 |  | 0.58164 | 9942 | 0.7508 |  |
|  | StxDe | 16 |  | 4.6386 | 9942 | 0.0001 |  |
|  |  |  |  |  |  |  |  |
| *napA3* | St | 2 | 12.445 | 8.6835 | 280 | 0.0406 | ns |
|  | De | 8 |  | 10.243 | 9942 | 0.0001 | 0^a^ 1^a^ 2^ab^ 3^a^ 4^a^ 6^a^ 10^b^ 14^ab^ 18^ab^ |
|  | Co(St) | 6 |  | 2.1294 | 9943 | 0.0649 |  |
|  | StxDe | 16 |  | 1.4914 | 9935 | 0.1402 |  |
|  |  |  |  |  |  |  |  |
| *narG1* | St | 2 | 5.3527 | 19.646 | 280 | 0.0035 | **0**: H^a^, A^b^, B^b^; **1,15**: H^a^, A^a^, B^b^; **2,18**: H^a^, A^b^, B^c^; **3,6**: ns; **4**: H^a^, A^b^, B^ab^; **10**: H^a^, A^ab^, B^b^ |
|  | De | 8 |  | 5.3856 | 9939 | 0.0002 | **H**: 0^ab^ 1^a^ 2^b^ 3^ab^ 4^ab^ 6^ab^ 10^ab^ 14^ab^ 18^ab^; **A**: ns; **B**:ns |
|  | Co(St) | 6 |  | 2.2101 | 9953 | 0.024 |  |
|  | StxDe | 16 |  | 3.4417 | 9930 | 0.0006 |  |
|  |  |  |  |  |  |  |  |
| *narG2* | St | 2 | 2.5306 | 136.82 | 280 | 0.0039 | **0,1,3,10,15, 18**: H^a^, A^b^, B^c^ ; **2,4**: H^a^, A^b^, B^b^ ; **6**: H^a^, A^b^, B^ab^ |
|  | De | 8 |  | 27.264 | 9948 | 0.0001 | **H**: 0^a^ 1^ab^ 2^ab^ 3^ab^ 4^ab^ 6^ab^ 10^ab^ 14^ab^ 18^b^; **A**: ns; **B**: 0^a^ 1^a^ 2^ab^ 3^a^ 4^ab^ 6^ab^ 10^b^ 14^b^ 18^b^ |
|  | Co(St) | 6 |  | 1.7486 | 9945 | 0.0687 |  |
|  | StxDe | 16 |  | 16.319 | 9922 | 0.0001 |  |
|  |  |  |  |  |  |  |  |
| *nrfA* | St | 2 | 5.4774 | 33.47 | 280 | 0.004 | **0, 10,14, 18**: H^a^, A^b^, B^c^ ; **1**: H^a^, A^a^, B^b^ ; **2**: H^a^, A^b^, B^b^, **3,6**:ns ; **4**: H^a^, A^b^, B^ab^ ; |
|  | De | 8 |  | 11.141 | 9927 | 0.0001 | **H**: 0^ac^ 1^ab^ 2^ac^ 3^abc^ 4^ac^ 6^c^ 10^abc^ 14^abc^ 18^bc^ ; **A**: ns; **B**: 0^a^ 1^a^ 2^a^ 3^ab^ 4^ab^ 6^ab^ 10^b^ 14^b^ 18^b^ |
|  | Co(St) | 6 |  | 2.2158 | 9947 | 0.0023 |  |
|  | StxDe | 16 |  | 5.023 | 9933 | 0.0001 |  |
|  |  |  |  |  |  |  |  |
| *nirSe* | St | 2 | 9.3271 | 49.029 | 280 | 0.0315 | H^a^, A^a^, B^b^ |
|  | De | 8 |  | 10.283 | 9945 | 0.0001 | 0^ab^ 1^ab^ 2^ab^ 3^ab^ 4^ab^ 6^a^ 10^abc^ 14^b^ 18^b,^ |
|  | Co(St) | 6 |  | 0.8263 | 9942 | 0.5627 |  |
|  | StxDe | 16 |  | 1.8479 | 9931 | 0.0513 |  |
|  |  |  |  |  |  |  |  |
| *nirSm* | St | 2 | 10.438 | 34.332 | 276 | 0.003 | **0, 1**: H^a^, A^b^, B^c^ ; **2, 3, 4**: H^a^, A^b^, B^ab^ ; **6, 18**: H^a^, A^ab^, B^b^ ; **10**: H^a^, A^b^, B^b^; **15**:ns |
|  | De | 8 |  | 5.0014 | 9935 | 0.0002 | **H**: ns; **A**: 0^a^ 1^a^ 2^a^ 3^a^ 4^ab^ 6^ab^ 10^b^ 14^ab^ 18^ab^; **B**: ns |
|  | Co(St) | 6 |  | 2.2158 | 9947 | 0.0445 |  |
|  | StxDe | 16 |  | 2.5049 | 9913 | 0.0066 |  |
|  |  |  |  |  |  |  |  |
| *nirSn* | St | 2 | 9.3256 | 75.304 | 280 | 0.0036 | H^a^, A^b^, B^c^ |
|  | De | 8 |  | 4.8463 | 9942 | 0.0005 | 0^a^ 1^a^ 2^a^ 3^ab^ 4^ab^ 6^ab^ 10^ab^ 14^b^ ^1^8^ab^ |
|  | Co(St) | 6 |  | 1.8308 | 9955 | 0.1139 |  |
|  | StxDe | 16 |  | 1.3509 | 9920 | 0.2104 |  |
