# Supplementary material for: Nitrate Reduction Functional Genes and Nitrate Reduction Potentials Persist in Deeper Estuarine Sediments. Why?
Source: PLoS One. 2014 Apr 11;9(4):e94111. doi: 10.1371/journal.pone.0094111 (PMC3984109; doi:10.1371/journal.pone.0094111)
Supplement: Table S6 — Pyrosequencing analysis results. Total number of sequences and percentage of sequences at different depths along the Colne estuary in June 2007. H: Hythe, A: Alresford, B: Brightlingsea. Numbers (0, 1, 2, 3, 4, 6, 10, 14 and 18) represent upper limit of depth layers. Values in bold represent contributions above 1% of the sequences in the sample. (DOCX) [file pone.0094111.s006.docx]

**Table S6.** **Pyrosequencing analysis results**. Total number of sequences and percentage of sequences at different depths along the Colne estuary in June 2007. H: Hythe, A: Alresford, B: Brightlingsea. Numbers (0, 1, 2, 3, 4, 6, 10, 14 and 18) represent upper limit of depth layers. Values in bold represent contributions above 1% of the sequences in the sample.

| **Sample** | **H0** | **H1** | | **H2** | | **H4** | | **H6** | | **H10** | | **H14** | | **H18** | | **A0** | | **A1** | | **A2** | | **A4** | | **A6** | | **A10** | | **A14** | | **A18** | | **B0** | | **B1** | | **B2** | | **B4** | | **B6** | | **B10** | | **B14** | | **B18** | |
| --- | --- | --- | --- | --- | --- | --- | --- | --- | --- | --- | --- | --- | --- | --- | --- | --- | --- | --- | --- | --- | --- | --- | --- | --- | --- | --- | --- | --- | --- | --- | --- | --- | --- | --- | --- | --- | --- | --- | --- | --- | --- | --- | --- | --- | --- | --- | --- |
| Total number of sequences | 1715 | 3025 | | 1675 | | 2084 | | 2112 | | 3940 | | 2387 | | 2166 | | 2016 | | 2043 | | 1221 | | 1620 | | 1440 | | 1240 | | 3282 | | 2510 | | 5053 | | 5500 | | 4291 | | 5593 | | 3244 | | 3362 | | 4994 | | 4466 | |
| **Phylum/ Class/ Order/Genus** | **Percentage of sequences (%)** | | | | | | | | | | | | | | | | | | | | | | | | | | | | | | | | | | | | | | | | | | | | | | |
| **Acidobacteriales** | 0.3 | | **1.1** | | **1.2** | | **1.2** | | **1.6** | | **2.7** | | **4.0** | | **3.3** | | **1.7** | | **2.2** | | **2.1** | | **3.0** | | **2.3** | | **3.4** | | **3.5** | | **4.2** | | **1.6** | | **2.4** | | 0.02 | | **4.7** | | **3.4** | | 0.9 | | 0.4 | | 0.7 |
| **Actinobacteria** | 0.4 | | 0.7 | | 0.6 | | 0.54 | | 0.8 | | 0.5 | | 0.64 | | 0.7 | | 0.14 | | 0.44 | | **1.7** | | 0.3 | | 0.2 | | 0.3 | | 0.2 | | 0.2 | | 0.5 | | 0.6 | | 0.8 | | 0.54 | | 0.6 | | 0.02 | | 0.06 | | 0.33 |
| **Fibrobacteres** | 0.1 | | 0 | | 0 | | 0 | | 0 | | 0 | | 0 | | 0 | | 0 | | 0 | | 0 | | 0 | | 0 | | 0 | | 0 | | 0 | | 0 | | 0 | | 0 | | 0 | | 0 | | 0 | | 0 | | 0 |
| **Actinomycetales** | 0.1 | | 0 | | 0 | | 0 | | 0 | | 0.1 | | 0 | | 0.04 | | 0.04 | | 0 | | 0 | | 0 | | 0.1 | | 0 | | 0.1 | | 0 | | 0 | | 0 | | 0 | | 0.01 | | 0 | | 0 | | 0 | | 0.1 |
| **Bacteroidetes;** | **14.8** | | **15.83** | | **15.3** | | **12.7** | | **12.3** | | **7.7** | | **5.8** | | **5.0** | | **8.5** | | **9.2** | | **8.1** | | **7.5** | | **8.0** | | **7.0** | | **4.1** | | **3.1** | | **7.6** | | **4.7** | | **5.0** | | **2.8** | | **2.9** | | 0.8 | | 0.2 | | 0.6 |
| **Cytophagales** | 0.5 | | 0.2 | | 0.3 | | 0.1 | | 0.2 | | 0.1 | | 0.04 | | 0.1 | | 0.1 | | 0 | | 0 | | 0 | | 0 | | 0 | | 0.03 | | 0 | | 0.1 | | 0.02 | | 0.1 | | 0.02 | | 0.03 | | 0 | | 0 | | 0 |
| **Flavobacteriale** | **2.0** | | **1.4** | | **1.4** | | **1.2** | | **1.4** | | 0.03 | | 0.02 | | 0.02 | | **1.1** | | **1.1** | | **1.1** | | **1.1** | | 0.8 | | 0.4 | | 0.5 | | 0.2 | | 0.6 | | 0.4 | | 0.7 | | 0.6 | | 0.4 | | 0.03 | | 0 | | 0 |
| **Firmicutes;** | 0.3 | | 0.4 | | 0.7 | | **1.0** | | 0.6 | | 0.2 | | 0.2 | | 0.4 | | 0.5 | | 0 | | 0 | | 0.06 | | 0.13 | | 0 | | 0.1 | | 0.1 | | 0.06 | | 0.1 | | 0.05 | | 0.5 | | 0.6 | | 0.06 | | 0.3 | | 0 |
| **Bacillales** | 0.1 | | 0.1 | | 0.1 | | 0 | | 0 | | 0 | | 0.1 | | 0 | | 0.3 | | 0.3 | | 0 | | 0.2 | | 0 | | 0 | | 0.1 | | 0.2 | | 0.2 | | 0.4 | | 0.4 | | 0.6 | | 0.4 | | 0.1 | | 0.14 | | 0.1 |
| **Lactobacillales;** |  | |  | |  | |  | |  | |  | |  | |  | |  | |  | |  | |  | |  | |  | |  | |  | |  | |  | |  | |  | |  | |  | |  | |  |
| **Lactobacillus spp.** | 0 | | 0 | | 0 | | 0 | | 0 | | 0 | | 0 | | 0 | | 0 | | 0 | | 0 | | 0 | | 0 | | 0 | | 0 | | 0 | | 0 | | 0 | | 0 | | 0 | | 0 | | 0.1 | | 0.04 | | 0 |
| **Chloroflexi** | **2.0** | | **2.8** | | **2.7** | | **4.7** | | **3.6** | | **5.2** | | **5.4** | | **6.2** | | **1.8** | | **1.6** | | **2.2** | | **2.8** | | **2.7** | | **3.2** | | **4.7** | | **6.4** | | **1.1** | | **1.2** | | **1.5** | | **2.8** | | **2.9** | | **1.2** | | 0.6 | | **1.9** |
| **Cyanobacteria** | 0.2 | | 0 | | 0 | | 0 | | 0.05 | | 0.03 | | 0 | | 0 | | 0.8 | | 0.2 | | 0.2 | | 0.1 | | 0 | | 0.08 | | 0 | | 0.03 | | 0.1 | | 0.2 | | 0.5 | | 0.1 | | 0.2 | | 0.2 | | 0 | | 0 |
| **Deferribacteres** | 0.1 | | 0.1 | | 0 | | 0.1 | | 0.1 | | 0.4 | | 0.4 | | 0.6 | | 0.1 | | 0.4 | | 0.3 | | 0.3 | | 0.4 | | 0.5 | | 0.4 | | **1.0** | | 0.2 | | 0.1 | | 0.2 | | 0.3 | | 0.3 | | 0.2 | | 0.1 | | 0.04 |
| **Deinococcales** | 0 | | 0 | | 0 | | 0 | | 0 | | 0 | | 0.04 | | 0 | | 0 | | 0.05 | | 0 | | 0 | | 0 | | 0 | | 0 | | 0 | | 0.02 | | 0 | | 0.02 | | 0 | | 0 | | 0 | | 0 | | 0 |
| **Nitrospira** | 0 | | 0.1 | | 0.1 | | 0 | | 0 | | 0.1 | | 0.04 | | 0.04 | | 0.1 | | 0.1 | | 0.2 | | 0.2 | | 0 | | 0 | | 0.1 | | 0.2 | | 0.3 | | 0.2 | | 0.3 | | 0.1 | | 0.3 | | 0.1 | | 0 | | 0.04 |
| **Proteobacteria; Alphaproteobacteria** | 0.2 | | 0.13 | | 0.1 | | 0.1 | | 0 | | 0.1 | | 0.1 | | 0.1 | | 0.2 | | 0.2 | | 0 | | 0.1 | | 0.1 | | 0 | | 0.2 | | 0.2 | | 0.2 | | 0.3 | | 0.2 | | 0.4 | | 0.2 | | 0 | | 0 | | 0.1 |
| **Rhizobiales** | 0 | | 0.01 | | 0 | | 0 | | 0.01 | | 0.1 | | 0 | | 0.01 | | 0.1 | | 0 | | 0 | | 0 | | 0 | | 0 | | 0 | | 0 | | 0.1 | | 0 | | 0 | | 0 | | 0 | | 0.1 | | 0.1 | | 0.14 |
| **Rhodobacterales** | 0.3 | | 0.4 | | 0.2 | | 0.2 | | 0.1 | | 0.4 | | 0.3 | | 0 | | 0.5 | | 0.1 | | 0.1 | | 0.5 | | 0.5 | | 0.2 | | 0.5 | | 0.8 | | **2.2** | | **1.3** | | **1.3** | | **1.0** | | 0.4 | | 0.1 | | 0 | | 0.3 |
| **Sphingomonadales;** |  | |  | |  | |  | |  | |  | |  | |  | |  | |  | |  | |  | |  | |  | |  | |  | |  | |  | |  | |  | |  | |  | |  | |  |
| **Erythrobacter spp.** | 0.1 | | 0.1 | | 0.1 | | 0 | | 0 | | 0 | | 0 | | 0 | | 0.1 | | 0 | | 0 | | 0 | | 0 | | 0 | | 0 | | 0 | | 0.1 | | 0 | | 0.1 | | 0.1 | | 0 | | 0.1 | | 0 | | 0 |
| **Proteobacteria; Betaproteobacteria** | 0.4 | | 0.6 | | 0.8 | | 0.3 | | 0.2 | | 0.7 | | 0.2 | | 0.7 | | 0.2 | | 0.1 | | 0.1 | | 0 | | 0.1 | | 0 | | 0.1 | | 0.2 | | 0 | | 0 | | 0 | | 0 | | 0 | | 0 | | 0 | | 0.1 |
| **Burkholderiales** | 0.3 | | 0.3 | | 0.4 | | 0.4 | | 0.3 | | 0.1 | | 0 | | 0.1 | | 0 | | 0 | | 0.1 | | 0 | | 0 | | 0 | | 0 | | 0 | | 0 | | 0 | | 0 | | 0 | | 0.1 | | 0 | | 0 | | 0.1 |
| **Rhodocyclales;** |  | |  | |  | |  | |  | |  | |  | |  | |  | |  | |  | |  | |  | |  | |  | |  | |  | |  | |  | |  | |  | |  | |  | |  |
| **Dechloromonas spp.** | 0.4 | | 0.1 | | 0 | | 0 | | 0.1 | | 0 | | 0 | | 0 | | 0.1 | | 0 | | 0.1 | | 0 | | 0 | | 0 | | 0.1 | | 0 | | 0 | | 0 | | 0 | | 0 | | 0 | | 0 | | 0 | | 0 |
| **Thauera spp.** | 0.2 | | 0.1 | | 0.1 | | 0 | | 0.1 | | 0.1 | | 0 | | 0.1 | | 0.3 | | 0.1 | | 0 | | 0 | | 0 | | 0 | | 0 | | **0** | | **0** | | **0** | | **0** | | **0** | | **0** | | **0** | | **0** | | **0** |
| **Proteobacteria; Deltaproteobacteria** | **29.4** | | **32.5** | | **31.9** | | **33.0** | | **33.7** | | **38.6** | | **39.5** | | **36.9** | | **36.7** | | **36.5** | | **37.9** | | **38.3** | | **37.7** | | **38.0** | | **35.9** | | **31.7** | | **29.6** | | **35.7** | | **35.4** | | **36.1** | | **34.7** | | **16.2** | | **10.5** | | **10.1** |
| **Desulfuromonadales**  **Desulfurobacterales** | 0.1  0.2 | | 0.1  0.1 | | 0  0.6 | | 0.1  0.6 | | 0.1  0.6 | | 0.2  0.5 | | 0.3  0.4 | | 0.2  0.3 | | 0.1  0.2 | | 0.3  0.2 | | 0.2  0.2 | | 0.4  0.4 | | 0.2  0.4 | | 0.2  0.2 | | 0.2  0.4 | | 0.2  0.6 | | 0.2  0.2 | | 0.3  0.6 | | 0.3  0.4 | | 0.3  0.2 | | 0.2  0.5 | | 0.1  0.1 | | 0  0 | | 0  0 |
| **Proteobacteria; Gammaproteobacteria** | 0.5 | | 0.7 | | 0.4 | | 0.8 | | 0.3 | | 0.7 | | 0.3 | | 0.2 | | **1.0** | | 0.7 | | 0.4 | | 0.6 | | **1.0** | | 0.3 | | 0.8 | | 0.6 | | **1.6** | | **1.8** | | **1.4** | | **1.0** | | 0.8 | | 0.2 | | 0 | | 0.05 |
| **Alteromonadales;** |  | |  | |  | |  | |  | |  | |  | |  | |  | |  | |  | |  | |  | |  | |  | |  | |  | |  | |  | |  | |  | |  | |  | |  |
| **Colwellia spp.** | 0 | | 0 | | 0 | | 0 | | 0 | | 0 | | 0 | | 0 | | 0.1 | | 0 | | 0.1 | | 0 | | 0.1 | | 0 | | 0 | | 0 | | 0.1 | | 0.1 | | 0 | | 0 | | 0 | | 0 | | 0 | | 0 |
| **Chromatiales;** |  | |  | |  | |  | |  | |  | |  | |  | |  | |  | |  | |  | |  | |  | |  | |  | |  | |  | |  | |  | |  | |  | |  | |  |
| **Rheinheimera spp.** | 0.1 | | 0.1 | | 0.1 | | 0.1 | | 0 | | 0 | | 0 | | 0 | | 0 | | 0 | | 0 | | 0 | | 0 | | 0 | | 0 | | 0 | | 0 | | 0 | | 0 | | 0 | | 0 | | 0 | | 0 | | 0 |
| **Enterobacteriales** | 0 | | 0.2 | | 0 | | 0 | | 0 | | 0.1 | | 0 | | 0 | | 0 | | 0.1 | | 0.1 | | 0 | | 0 | | 0 | | 0 | | 0 | | 0 | | 0 | | 0 | | 0 | | 0 | | 0 | | 0.3 | | 0.2 |
| **Legionellales;** |  | |  | |  | |  | |  | |  | |  | |  | |  | |  | |  | |  | |  | |  | |  | |  | |  | |  | |  | |  | |  | |  | |  | |  |
| **Coxiella spp.** | 0 | | 0 | | 0 | | 0 | | 0 | | 0 | | 0 | | 0 | | 0 | | 0.1 | | 0.1 | | 0 | | 0 | | 0.1 | | 0 | | 0 | | 0.1 | | 0 | | 0 | | 0.1 | | 0 | | 0 | | 0 | | 0.3 |
| **Methylococcales** | 0.4 | | 0.5 | | 0.3 | | 0.2 | | 0.2 | | 0.01 | | 0.1 | | 0.4 | | 0 | | 0.1 | | 0 | | 0.2 | | 0.1 | | 0 | | 0.1 | | 0 | | 0.01 | | 0.14 | | 0.2 | | 0.1 | | 0.1 | | 0.1 | | 0.2 | | 0.1 |
| **Pseudomonadales** | **2.6** | | **2.0** | | **1.2** | | **1.7** | | **1.5** | | **1.1** | | 0.8 | | 0.4 | | **4.1** | | **4.7** | | **3.9** | | **4.5** | | 0.3 | | 0 | | 0.2 | | 0.04 | | 0.3 | | 0.3 | | 0.3 | | 0.2 | | 0.1 | | 0.2 | | 0.4 | | 0.8 |
| **Thiotricales;** |  | |  | |  | |  | |  | |  | |  | |  | |  | |  | |  | |  | |  | |  | |  | |  | |  | |  | |  | |  | |  | |  | |  | |  |
| **Cycloclasticus spp.** | 0 | | 0 | | 0 | | 0 | | 0 | | 0 | | 0 | | 0 | | 0 | | 0 | | 0 | | 0 | | 0 | | 0 | | 0 | | 0 | | 0.05 | | 0 | | 0 | | 0 | | 0 | | 0 | | 0 | | 0 |
| **Xanthomonadales;** |  | |  | |  | |  | |  | |  | |  | |  | |  | |  | |  | |  | |  | |  | |  | |  | |  | |  | |  | |  | |  | |  | |  | |  |
| **Stenotrophomonas spp.** | 0 | | 0 | | 0 | | 0 | | 0 | | 0 | | 0 | | 0.1 | | 0 | | 0 | | 0 | | 0 | | 0 | | 0 | | 0 | | 0 | | 0.1 | | 0.1 | | 0 | | 0 | | 0.1 | | 0 | | 0 | | 0 |
| **Thermomonas spp.** | 0 | | 0 | | 0 | | 0 | | 0 | | 0.1 | | 0 | | 0 | | 0 | | 0 | | 0 | | 0 | | 0 | | 0 | | 0 | | 0 | | 0 | | 0 | | 0 | | 0 | | 0 | | 0 | | 0 | | 0 |
| **Vibrionales;** |  | |  | |  | |  | |  | |  | |  | |  | |  | |  | |  | |  | |  | |  | |  | |  | |  | |  | |  | |  | |  | |  | |  | |  |
| **Vibrio spp.** | 0 | | 0.1 | | 0.1 | | 0 | | 0 | | 0.1 | | 0.1 | | 0.1 | | 0. | | 0.1 | | 0 | | 0 | | 0 | | 0 | | 0 | | 0 | | 0 | | 0 | | 0 | | 0 | | 0 | | 0 | | 0 | | 0 |
| **Planctomycetes** | 0.8 | | **1.0** | | 0 | | 0.3 | | 0.7 | | 0.5 | | 0.3 | | 0.4 | | **1.2** | | 0.7 | | **1.2** | | 0.9 | | **1.7** | | 0.6 | | 0.7 | | 0.5 | | **1.4** | | **1.8** | | **1.0** | | **1.5** | | **1.5** | | **1.1** | | **1.5** | | **1.3** |
| **Fusobacteria** | 0 | | 0 | | 0 | | 0 | | 0 | | 0 | | 0 | | 0 | | 0 | | 0 | | 0 | | 0 | | 0 | | 0.1 | | 0 | | 0 | | 0 | | 0 | | 0 | | 0 | | 0 | | 0 | | 0 | | 0 |
| **Fusobacteriales;** |  | |  | |  | |  | |  | |  | |  | |  | |  | |  | |  | |  | |  | |  | |  | |  | |  | |  | |  | |  | |  | |  | |  | |  |
| **Leptotrichia spp.** | 0 | | 0 | | 0 | | 0 | | 0 | | 0 | | 0 | | 0 | | 0 | | 0 | | 0 | | 0 | | 0 | | 0 | | 0 | | 0 | | 0 | | 0 | | 0 | | 0 | | 0 | | 0 | | 0.1 | | 0 |
| **Flavobacteriales** | 0.1 | | 0.1 | | 0 | | 0 | | 0 | | 0 | | 0.1 | | 0 | | 0.1 | | 0.1 | | 0.2 | | 0.1 | | 0.1 | | 0.3 | | 0.1 | | 0 | | 0.1 | | 0 | | 0.2 | | 0 | | 0.1 | | 0 | | 0 | | 0 |
| **Verrucomicrobia** | **1.2** | | **2.0** | | **1.2** | | **2.5** | | **1.3** | | 0.9 | | 0.8 | | 0.9 | | 0.7 | | 1.2 | | 0.6 | | 0.8 | | 0.7 | | **1.1** | | 0.7 | | 0.3 | | **1.8** | | 0.8 | | 0.2 | | 0.7 | | 0 | | 0 | | 0 | | 0 |
| **Fusobacteria** | 0.1 | | 0.1 | | 0 | | 0 | | 0.1 | | 0 | | 0.1 | | 0.1 | | 0 | | 0 | | 0 | | 0 | | 0 | | 0 | | 0 | | 0 | | 0 | | 0 | | 0 | | 0 | | 0 | | 0 | | 0 | | 0 |
| **Gemmatimonadetes** | 0 | | 0 | | 0 | | 0 | | 0 | | 0 | | 0.1 | | 0 | | 0 | | 0 | | 0 | | 0 | | 0 | | 0.1 | | 0 | | 0 | | 0 | | 0 | | 0 | | 0 | | 0.1 | | 0 | | 0 | | 0 |
| **Spirochaetes** | 0.2 | | 0.6 | | 0.8 | | 0.4 | | 0.5 | | 0.8 | | 0.7 | | 0.9 | | 0.2 | | 0.4 | | 0.2 | | 0.1 | | 0.2 | | 0.3 | | 0.6 | | 0.6 | | 0.4 | | 0.6 | | 0.5 | | 0.6 | | 0.5 | | 0.3 | | 0.1 | | 0.04 |
| **Marine bacterium** | 0.6 | | 0.4 | | 0.4 | | 0.2 | | 0.6 | | 0.1 | | 0.2 | | 0.2 | | 0.8 | | 0.7 | | 0.4 | | 0.2 | | 0.6 | | 0.2 | | 0.3 | | 0.2 | | 0.7 | | 0.6 | | 0.4 | | 0.2 | | 0.2 | | 0 | | 0 | | 0 |
| **Uncultured bacteria** | **40.9** | | **35.03** | | **38.9** | | **37.56** | | **38.94** | | **37.73** | | **38.92** | | **41.49** | | **38.12** | | **38.01** | | **37.2** | | **37.34** | | **41.57** | | **43.42** | | **45.27** | | **48.43** | | **48.46** | | **45.24** | | **48.51** | | **44.43** | | **47.97** | | **77.66** | | **84.96** | | **82.49** |
